# Acetylation of the histone-like protein HBsu at specific sites alters gene expression during sporulation in *Bacillus subtilis*

**DOI:** 10.3389/fmicb.2025.1629989

**Published:** 2025-10-23

**Authors:** Liya Popova, Hritisha Pandey, Olivia R. Schreiber, Charalampos Papachristou, Valerie J. Carabetta

**Affiliations:** ^1^Department of Biomedical Sciences, Cooper Medical School of Rowan University, Camden, NJ, United States; ^2^Department of Mathematics, Rowan University, Glassboro, NJ, United States

**Keywords:** post-translational modification, PTM, spore, acetyl, HU, lysine acetylation, bacteria

## Abstract

**Introduction:**

Sporulation is an adaptive response to starvation in bacteria that consists of a series of developmental changes in cellular morphology and physiology, leading to the formation of a highly resistant endospore. In *Bacillus subtilis*, there is an intricate developmental program which involves the precise coordination of gene expression and ongoing morphological changes to yield the mature spore. The histone-like protein HBsu is involved in proper spore packaging and compaction of the chromosomal DNA.

**Methods:**

Previously, we found that the acetylation of different lysine residues on HBsu impairs sporulation frequency and spore resistance properties. One mechanism by which HBsu influences the process of sporulation could be through the regulation of gene expression. To test this idea, we performed RT-qPCR to analyze gene expression throughout the sporulation process in wildtype and seven acetylation-mimicking (lysine to glutamine substitutions) mutant strains.

**Results:**

Acetylation of HBsu at K41 increased the expression of key early and late sporulation genes, especially during the later stages. For example, overexpression σ^F^ and σ^G^ drive expression of their regulon members at inappropriate times. The gene expression profiles for the acetyl-mimic mutants at K3, K37, K75, K80, and K86 were largely unchanged, but did have reductions of key late sporulation proteins, which could explain the observed defects in spore resistance properties.

**Discussion:**

These findings suggest that K41 acetylation activates gene expression and might represent an “on–off” switch for important regulatory factors as cells transition from early to late phases. We propose that the acetylation of HBsu at specific sites regulates gene expression during sporulation and this is required for proper timing and coordination.

## 1 Introduction

Sporulation is an adaptive response to starvation in bacteria that consists of a series of developmental changes in cellular morphology and physiology, leading to the packaging of one cell into a highly resistant spore (McKenney et al., 2013; Price et al., 2012; Tan and Ramamurthi, 2014). The process can be divided into seven stages based on morphology (Errington, 1993). It is important to note that the sporulation stages are usually described as discrete morphological events, whereas the process is continuous and several steps occur concomitantly (Chai et al., 2025). No morphological changes at stage 0 of sporulation occur within the cell, but the decision to sporulate has already been made with high levels of the activated form of the master transcriptional regulator Spo0A (Stragier and Losick, 1996). Activation of Spo0A occurs through a multistep phosphorelay (Fujita et al., 2005; Fujita and Losick, 2005) and variation of phosphorylated Spo0A (Spo0A ~ P) levels influences cell development (Piggot and Hilbert, 2004). The transition state is characterized by the initial activation of Spo0A at the end of exponential growth (Stragier and Losick, 1996). An increase in Spo0A ~ P levels and activation of the sigma factor σ^H^ directs cells into the sporulation program that is later driven by the compartment-specific master regulators of early sporulation σ^F^ and σ^E^, forespore and mother cell specific, respectively (Fimlaid and Shen, 2015). The production of σ^F^ occurs before septation, which becomes active after septation in the forespore (Arigoni et al., 1996; Gholamhoseinian and Piggot, 1989; Stragier and Losick, 1996). The σ^F^ activation cascade includes SpoIIE, SpoIIAB, and SpoIIAA proteins, which are synthesized in the pre-divisional cell (Duncan and Losick, 1993; Min et al., 1993). σ^F^ is inactive when bound with the anti-sigma factor SpoIIAB. SpoIIAA is an anti-anti-sigma factor that relieves the repression of SpoIIAB on σ^F^ activity (Duncan et al., 1995). SpoIIE is a serine phosphatase (Levdikov et al., 2012) that catalyzes the dephosphorylation of SpoIIAA, thereby contributing to the forespore-specific activation of σ^F^ (Arigoni et al., 1996; Carniol et al., 2005). The expression of σ^F^-dependent genes is transcriptionally regulated by *rsfA*, a DNA-binding protein that blocks the premature appearance of σ^E^ (Juan Wu and Errington, 2000). While σ^F^ is activated in the forespore, σ^E^ in the mother cell is activated after septation is complete (Stragier and Losick, 1996). Pro-σ^E^ is synthesized before the asymmetric division from the Spo0A ~ P dependent operon *spoIIG* (Ju et al., 1997). SpoIIGA is a protease that cleaves pro-σ^E^ and activates it (Imamura et al., 2008; McBride and Haldenwang, 2004).

Upon activation of both sigma factors, entry into sporulation becomes irreversible (Dworkin and Losick, 2005; Narula et al., 2012). Activation of the compartment-specific sigma factors σ^F^ and σ^E^ is initiated by asymmetric division of the cell (Stragier and Losick, 1996). Morphologically, during stage I of sporulation, the chromosome is replicated and extended from one pole to the other, forming a structure called the axial filament (Schumacher et al., 2016). The formation of asymmetric septa occurs during stage II of sporulation. During stage III, the polar septum between the mother cell and the forespore curves around the forespore, leading to the engulfment of the forespore (Errington, 1993). The early engulfment stage is primarily driven by proteins synthesized in the mother cell, SpoIID, SpoIIM, SpoIIP, which are under the control of σ^E^ (Doan et al., 2005; Gutierrez et al., 2010). Activation of σ^G^ in the forespore, followed by activation of σ^K^ in the mother cell, signals the transition of the sporulation to stage IV (Stragier and Losick, 1996; Sun et al., 1991). Morphologically, the mother cell produces the proteinaceous coat that further assembles around the forespore (Driks and Eichenberger, 2016; McKenney and Eichenberger, 2012). Similarly to σ^F^, σ^G^ is held inactive by SpoIIAB in the pre-engulfment forespore, and the activation of σ^G^ requires its release (Chary et al., 2005; Serrano et al., 2004). Activation of σ^G^ in the forespore is followed by activation of the mother cell specific σ^K^ (Serrano et al., 2004; Stragier and Losick, 1996). Transcription of *sigK* occurs in the mother cell from a σ^E^-dependent promoter (Hilbert and Piggot, 2004). Similarly to σ^E^, σ^K^ appears initially as pro-σ^K^ approximately 1 h before the process, depending on the gene expression in the prespore (Hilbert and Piggot, 2004). The σ^G^ regulon includes *ssp* genes, which encode the small acid soluble proteins (SASPs), late sporulation genes, and genes that control germination (Moeller et al., 2009; Nerber and Sorg, 2024; Setlow and Setlow, 1993; Wang et al., 2006). The σ^K^ regulon directs transcription of 14 spore coat genes, including *cotD* and *spoVK* (Haldenwang, 1995). Stage V of sporulation is characterized by the forespore depositing a protective exterior consisting of two layers of peptidoglycan, the primordial germ cell wall, and the cortex (Meador-Parton and Popham, 2000). This is followed by stage VI, which is the final maturation of the spore (Tan and Ramamurthi, 2014). The late stages of sporulation include spore DNA coating with SASPs, which protects spores from heat and UV radiation, and packaging of dipicolinic acid inside the spore core, to protect against heat and desiccation (Higgins and Dworkin, 2012; Ross and Setlow, 2000). The mature spore is released during the final stage VII through the lysis of the mother cell (Piggot and Hilbert, 2004; Stragier and Losick, 1996; Tan and Ramamurthi, 2014).

The mature spore possesses phenomenal resistance properties, allowing for survival under extreme environmental conditions, such as exposure to heat, radiation, antibiotics, disinfectants, and lack of nutrients (Cortesao et al., 2019; Setlow, 2014). The structural protection of the spore chromosome from such insults is one reason for these properties, which is dependent upon the activity of the SASPs (Magge et al., 2008; Mason and Setlow, 1986; Moeller et al., 2008; Moeller et al., 2009). Previously, the histone-like protein HBsu was implicated to play a role in condensation of the spore chromosome, in addition to the SASPs (Ross and Setlow, 2000). HBsu colocalizes with the SASPs on the DNA, in both the mother cell and spore, and counteracts SASP-mediated changes in persistence length and supercoiling (Ross and Setlow, 2000). It was proposed that HBsu counteracting the SASP-mediated increase in persistence length might be required for chromosome packaging during sporulation. So far, it is known that HBsu binds non-specifically to DNA and regulates DNA compaction, replication initiation, recombination, and repair (Karaboja and Wang, 2022; Kohler and Marahiel, 1997; Fernández et al., 1997).

Our lab is interested in understanding the regulatory role of HBsu acetylation. N^Ɛ^-lysine acetylation is a ubiquitous regulatory post-translational modification (PTM), that can influence protein conformation and interactions with substrates, leading to change of function (Christensen et al., 2019; Luu and Carabetta, 2021; Luu et al., 2021; Carabetta and Cristea, 2017). It is hypothesized that the relationship between acetylation and central metabolism may be regulated by the cellular response to nutrient availability or the energy state of the cell (Castano-Cerezo et al., 2014). Over the last 5 years, acetylomes and other PTMs have been characterized in more than 20 bacterial species, and over 50 bacterial acetylomes have been reported from 2008 to 2019 (Christensen et al., 2019; Popova et al., 2024; Carabetta and Cristea, 2017). Previously, we identified seven acetylation sites on HBsu (Carabetta et al., 2016) and found that acetylation at some residues reduced the DNA binding affinity and regulated nucleoid compaction (Carabetta et al., 2019). Furthermore, we investigated whether the acetylated state of HBsu can influence the process of sporulation and the resistance properties of mature spores (Luu et al., 2021). Using glutamine substitutions to mimic the acetylated state and arginine substitutions to mimic the unacetylated state, we found that certain mutants led to a reduction in sporulation frequency and resistance to heat, formaldehyde, and ultraviolet (UV) light (Luu et al., 2021). We proposed that acetylation at key residues of HBsu is required for proper sporulation. However, the exact underlying mechanism by which HBsu acetylation regulates this process is unknown.

**Figure 1:**
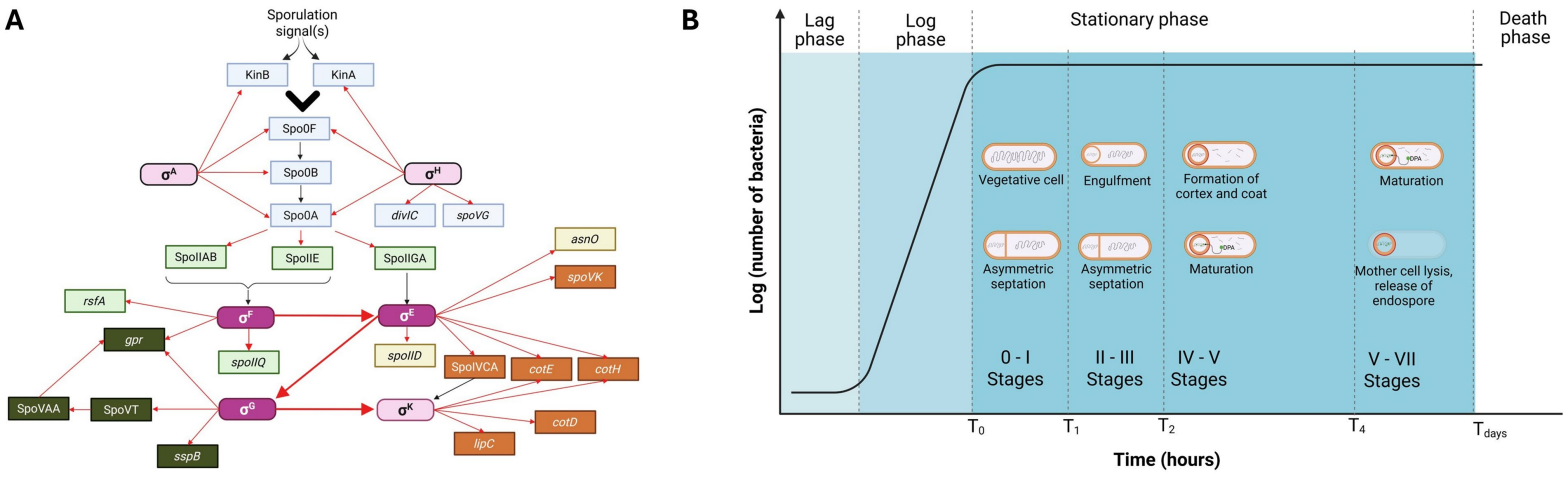
**(A)** Interplay of sporulation-specific σ factors in *B. subtilis.* The network displays the interactions among the genes, and their products, that were analyzed in this study. The sigma factors that were directly assayed are dark pink, and the others (light pink) were assayed for activity by monitoring expression of their regulon members. The factors that work before asymmetric septation are in light blue. The early sporulation genes that are forespore-specific are colored in light green, the mother cell-specific are in light yellow, the late sporulation forespore-specific are in dark green, the mother-cell specific late sporulation genes are in orange. Nodes connected by red arrows represent a transcriptional relationship, and those connected by black arrows represent post-translational regulation. The large arrows represent sigma factor cross-compartment signaling. **(B)** Growth curve of *Bacillus subtilis* during sporulation. The studied timepoints and schematic representation of the corresponding sporulation stages are indicated. Created in BioRender. Carabetta, V. (2025) https://BioRender.com/izhtbnu and /0t4n57w.

We hypothesized that one mechanism by which acetylation of HBsu could influence sporulation is by the regulation of sporulation-specific gene expression. To test this hypothesis, we selected important sporulation-specific genes and regulatory factors (Figure 1A) that are turned on throughout the sporulation processes and examined expression by quantitative real-time PCR (qRT-PCR). We found that the *hbsK41Q* mutant was different from the other mutants, in that the expression of 17/25 genes analyzed were significantly upregulated at the later sporulation timepoints, whereas most of the other mutant strains exhibited the opposite phenotype. *hbsK3Q, hbsK37Q, hbsK75Q, hbsK80Q, hbsK86Q* strains had downregulated several important genes during late sporulation. Based on these findings, we propose that acetylation at K41 plays a significant regulatory role during sporulation, which might be responsible for switching on or off gene expression as the cell moves from the early to late developmental stages.

## 2 Materials and methods

### 2.1 Bacterial strains, media, and growth conditions

All *B. subtilis* strains are listed in Table 1. The *hbs* point mutations were previously constructed at the native locus, as previously described (Carabetta et al., 2019). LB (Luria-Bertani) agar and Schaeffer’s sporulation media (DSM) were prepared as described previously (Albano et al., 1987). Strains were streaked out on LB plates and incubated overnight at 30 °C. The following morning, cells were inoculated into 20 mL of DSM and grown at 37 °C with aeration for 6 h, with growth monitored by Klett colorimetry. 1 mL of cells were harvested after 2, 3, 4, and 6 h, corresponding to the entry into stationary phase (T_0_), and hours after (T_1_, T_2_, and T_4_ time points), which correlates to stages of sporulation. The samples were centrifuged at 13,000 *x g* for 3 min. The resulting cell pellets were processed further either for RT-qPCR, western blot, or microscopy.

**Table 1:** Strains used in this study.

*B. subtilis* strain number	Genotype*	References
BD630	*his leu8 metB5*	Laboratory strain
BD7493	*hbsK86Q*	Carabetta et al. (2019)
BD8119	*hbsK37Q*	Carabetta et al. (2019)
BD8147	*hbsK41Q*	Carabetta et al. (2019)
BD8148	*hbsK41R*	Carabetta et al. (2019)
BD8219	*hbsK18Q*	Carabetta et al. (2019)
BD8398	*hbsK75Q*	Carabetta et al. (2019)
BD8576	*hbsK80Q*	Carabetta et al. (2019)
BD8577	*hbsK3Q*	Carabetta et al. (2019)

*All strains are in the BD630 background.

### 2.2 Quantitative real-time PCR

Samples collected at each timepoint were processed with using the FastRNA™ Pro Blue Kit (MP Biomedicals) to extract total RNA as per manufacturer’s instructions, with modifications. Briefly, the cell pellets were resuspended in 1 mL RNApro solution and lysed in tubes containing Lysing Matrix B provided in the kit. The sample tubes were processed using the FastPrep-25 5G Instrument (MP Biomedicals) for three cycles to ensure lysis, 45 s for the first two, then one 30 s cycle. The concentration of total RNA was measured using a NanoDrop 2000 spectrophotometer (Thermo Fisher Scientific). Average RNA concentrations obtained varied from 800 to 3,500 ng/μL, with an A260: A280 ratio between 1.8 and 2.1. Next, RNA samples were treated using the Turbo DNA-*free*™ Kit (Invitrogen) according to the manufacturer’s instructions, to remove potential contaminating genomic DNA. Reverse transcription was carried out using the SuperScript™ IV VILO™ kit (Invitrogen) as per manufacturer’s instructions, with the following modifications. The annealing time was extended to 20 min at 25 °C and the reverse transcription time to 20 min at 50 °C. The resulting cDNA was purified using the MinElute PCR Purification Kit (Qiagen) according to the manufacturer’s protocol with the following modification. To elute DNA, 12 μL of elution buffer was added to the spin column containing sample, incubated for 5 min at 37 °C, and then centrifuged at 12,000 *x g* for 2 min. The cDNA samples were diluted 1:5 in nuclease-free water (1X concentration) and the concentrations measured using the NanoDrop 2000. For standard curves, the cDNA was diluted 5-fold six times. Five mL of 1X, 0.2X, 0.04X, 0.008X, 0.0016X, and 0.00032X concentrations of cDNA for standards and 5 mL of 0.1X for samples, were added to a MicroAmp Optical 96-well plate (Applied Biosystems) 0.12.5 μL of PowerTrack SYBR Green (Applied Biosystems) and 0.25 μL of each primer (10 μM) were added to each well, for a 25 μL total volume. All primers used in this study are listed in Supplementary Table S1. The primers were designed using Primer3 (version 4.1.0), and synthesized by Eton Biosciences (Union, NJ). Plates were run on QuantStudio 7 Pro Real-Time PCR instrument (Applied Biosystems) and analyzed using the relative standard curve method by the built in QuantStudio Design and Analysis software (Applied Biosystems). A melt curve analysis was performed after each run. All reactions were run in technical duplicates, and each experiment was repeated at least three independent times. The *rpoD* transcript was used as an internal control to adjust for differing amounts of input cDNA. All data was first normalized to the corresponding strain and time point input RNA value determined for *rpoD* and then the mutants compared to the wildtype normalized RNA value of the corresponding time point (Larionov et al., 2005). A one-way ANOVA for each gene at each timepoint was analyzed to identify significant differences among strains, followed by a *post hoc* Benjamini–Hochberg correction and Dunnett’s test to control for multiple comparisons. A *p*-value of < 0.05 was considered significant. Figures displaying normalized fold gene expression were created using GraphPad Prism (Version 9.4.0).

### 2.3 Western blot

One mL of cells was collected by centrifugation after 4 and 6 h of growth in DSM at 37 °C. The cell pellets were resuspended in STM buffer (0.71 mM MgCl_2_, 7.14 mM NaCl, 73 mM Sucrose, 35.7 mM Tris, pH8) + 350 μg/mL lysozyme with volumes normalized by 1/10th of the measured Klett value and incubated at 37 °C for 5 min. Samples were boiled 1X cracking buffer (0.225 M Tris–HCl, pH 6.8, 50% glycerol, 5% sodium dodecyl sulfate [SDS], 0.05% bromophenol blue, 1% β-mercaptoethanol) for 10 min and an equal amount of protein loaded on a 15% Tris-Glycine gel. Proteins were transferred to a nitrocellulose membrane using the iBlot 3 Western Blot Transfer System (Invitrogen), according to the manufacturer’s instructions. Membranes were blocked in 5% milk in Tris-buffered saline containing 1% Tween-20 (TBS-T) for 1 h and then incubated overnight at 4 °C with the indicated primary antibodies, at a 1:5,000 dilution for anti-SigF, anti-SigK, or anti-SpoIIAB, and a 1:10,000 dilution for anti-SigG. A 1:5,000 dilution of anti-EFG was included as a loading control. The anti-SigF and anti-SigG antibodies were kindly provided by D. Rudner (Harvard Medical School) and the anti-SigK and anti-SpoIIAB antibodies were kindly provided by N. Bradshaw (Brandeis University). The following day, the membranes were washed four times in TBS-T, then incubated in anti-rabbit secondary antibodies conjugated to horseradish peroxidase (Invitrogen) at a1:5000 dilution for 1 h at room temperature and washed in TBS-T as before. Bands were visualized using the ECL Prime Western Blotting Detection Reagent (Cytiva) according to the manufacturer’s instructions and imaged using the ChemiDoc™ MP Imaging System (BIO-RAD). All western blots were quantified using Image J (NIH), and all signal intensities were normalized EF-G. To calculate fold changes compared to wildtype, EF-G normalized intensities in the mutant strains were divided by the EF-G normalized intensities in wildtype.

### 2.4 Sporulation staging using phase-contrast microscopy

The cell cultures of BD630 and mutant strains were grown to T_0_, T_1_, T_2_, and T_4_ as described above. Five mL of culture was harvested and centrifuged at 11,000 *x g* for 3 min. The supernatant was discarded, and the cell pellets resuspended in 5 mL of phosphate buffered saline (PBS). Cells were washed once with 1 mL of PBS, centrifuged at 11,000 *x g* for 1 min, and fixed in 1 mL of 4% formaldehyde for 15 min at room temperature. Following incubation, pellets were washed twice with 1 mL of PBS, and permeabilized for 10 min in 500 μL of 25 μg/mL (final concentration) lysozyme, and then immediately washed three times with 1 mL of PBS. Fixed and permeabilized cells were stained for 15 min with 10 μg/mL of wheat germ agglutinin conjugated with Alexa Flour 488 (WGA), washed with 500 μL of PBS, for a total of three times, and then stained for 5 min with 2 μg/mL of Sytox to stain DNA. Following staining, cells were washed with PBS, resuspended in 500 μL of PBS, and 1 μL of cells was placed on 1% agarose pads, and imaged on a Nanoimager super-resolution microscope (ONI). Mature, phase-bright spores were assessed in transillumination mode. Sporulating cells were imaged using 488 nm laser. The laser power was set at 4–5% for the 488 nm wavelength for Sytox and 19–20% for WGA. Samples were analyzed using NimOS (version 1.19.4). Staging was determined using biological replicates, analyzing at least 200 cells in each sample.

### 2.5 Determination of sporulation frequency

The sporulation frequency was performed as previously described (Carabetta et al., 2013) with the following modifications. Samples were collected after 2 h of incubation in DSM (T_0_). The sporulation frequency was calculated as heat-resistant colony-forming units (CFUs) per ml/total viable cells (CFUs/ml). The experiments were carried out at least three independent times. The two-tail Student’s *t*-test was used to compare the sporulation frequency between samples. A *p*-value < 0.05 was considered significant.

## 3 Results

### 3.1 Selection of reporter genes and RT-qPCR assay development

We hypothesized that one way acetylation of HBsu influences the process of sporulation is through the regulation of gene expression. To test this idea, a quantitative real-time PCR (RT-qPCR) assay was developed and optimized. HBsu is acetylated at seven sites throughout the protein, at K3, K18, K37, K41, K75, K80, and K86 (Carabetta et al., 2016). To examine the influence of acetylation, we utilized our collection of point mutations at the native *hbs* locus that mimic the acetylated state [glutamine substitutions (Carabetta et al., 2019)]. We examined four different house-keeping genes to serve as an internal control, *rpoA*, *rrnA-16S*, *rrnA-5S*, and *rpoD*. The efficiency of each set of primers (Supplementary Table S2) was determined and *rpoD* was ultimately selected as the internal control for normalization of differing amounts of input cDNA. Based upon the expression patterns of sporulation-specific genes, 25 genes were selected for analysis (Stragier and Losick, 1996). The selected genes were representative of various early and late stages of sporulation. Samples were collected at 4 time points, T_0_–T_4_. The T_0_ time point corresponds to the beginning of sporulation (stages 0–I), when the cells “decide” to sporulate. The T_1_ time point corresponds to stages II–III of sporulation, characterized by significant changes in the cell morphology, including asymmetric septation. The T_2_ time-point is later in the process and corresponds to stages IV–VI of sporulation, while T_4_ corresponds to stages V–VII. As the sporulation-specific sigma factors are important master regulators driving this process, various regulon members were included as reporters of sigma factor activity (Supplementary Table S3 and Figure 1A). Additionally, we analyzed the asynchronous cultures at the selected time-points to determine and confirm the staging of sporulation (Figure 1B). As expected, there were cells at different stages concomitantly within the same population (Supplementary Figure S1).

### 3.2 Acetylation at lysine 41 increases expression of the early sporulation genes

**Figure 2:**
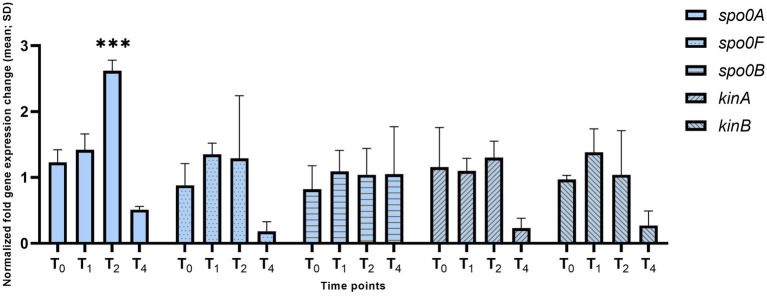
Expression of the phosphorelay genes in the *hbsK41Q* mutant. Wildtype and *hbsK41Q* mutant cells were grown in DSM for 6 h and analyzed at time points T_0_–T_4_. Values displayed are the mean fold change values of technical duplicates from at least three biological replicates with the standard deviation (SD). Adjusted *p*-values after correction for multiple comparisons are reported, ***p < 0.001.

The *hbsK41Q* mutant and wild-type strains were grown in DSM and analyzed throughout sporulation from T_0_–T_4_. First, we examined the activity of the housekeeping sigma factor σ^A^ and the stationary phase sigma factor σ^H^, which are responsible for the entry into the sporulation process, by examining the expression of the phosphorelay proteins (*kinA, kinB, spo0A, spo0B,* and *spo0F*). At T_0_ and T_1_, representing early sporulation when these genes are likely important, we did not observe significant differences in expression compared to the wild type (Figure 2). At T_2_, there was a 2.6-fold increase in *spo0A* expression (adj. *p*-value = 0.001). At T_4,_ there were reduced levels of *spo0F*, *spo0A, kinA*, and *kinB*, but these differences were not statistically significant and would likely not biologically relevant as phosphorylated Spo0A is not required at this late stage.

**Figure 3:**
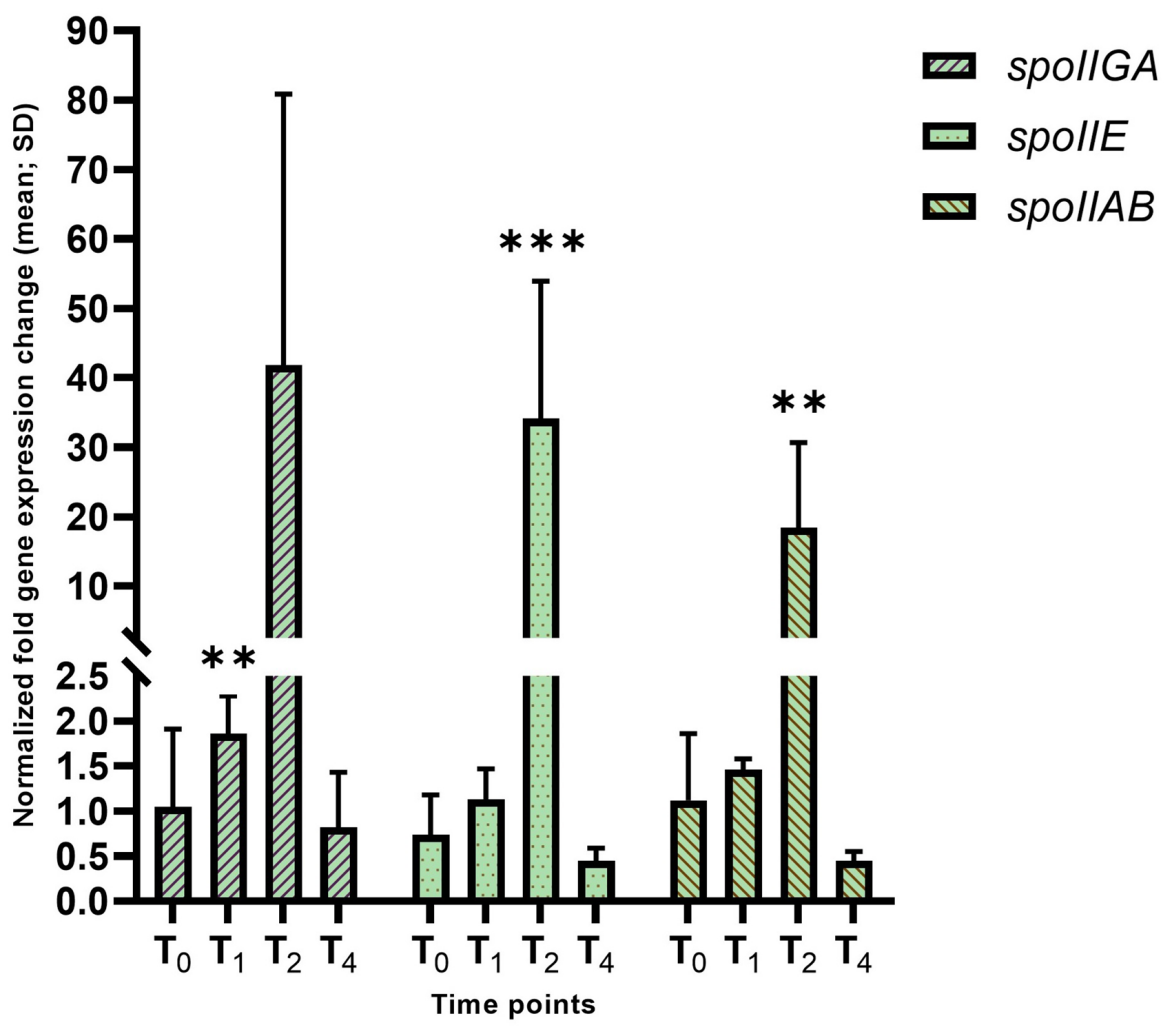
Dynamics of the transcription of early sporulation-specific reporters of Spo0A/σ^A^ and Spo0A/σ^H^ over T_1_–T_4_ timepoints in the *hbsK41Q* mutant. Wild-type and the *hbsK41Q* mutant cells were grown in DSM for 6 h and analyzed at time points T_0_–T_4_. Values displayed are the mean fold change values of technical duplicates from at least three biological replicates with the standard deviation (SD). Adjusted *p*-values after correction for multiple comparisons are reported, **p < 0.01, ***p < 0.001.

Next, we examined the expression of genes that are dependent upon Spo0A ~ P, including *spoIIE,* and the *spoIIA* and *spoIIG* operons, with the proteins required during stage II of sporulation (Figure 3). Expression of these genes typically occurs before asymmetric septation and they are all involved in the activation of σ^F^. *spoIIAB* was 18.4-fold (adj. *p*-value = 0.0074) increased at T_2_. Despite this increase in transcription, a corresponding increase in protein levels was not observed (Supplementary Figure S2). Similarly, there was a 1.86-fold overexpression of *spoIIGA* at T_1_ (adj. *p*-value = 0.0071) and 41.8-fold overexpression at T_2_. *spoIIE* was significantly overexpressed 34.1-fold (adj. *p*-value = 0.0018) at T_2_. This suggests that the increase in *spo0A* transcription observed at T_2_ leads to more Spo0A ~ P in the cell, which increases expression of Spo0A-dependent promoters, especially at T_2_. In agreement, bursts in *spo0A* transcription have been observed to result in increases in Spo0A ~ P levels (Mirouze et al., 2011).

**Figure 4:**
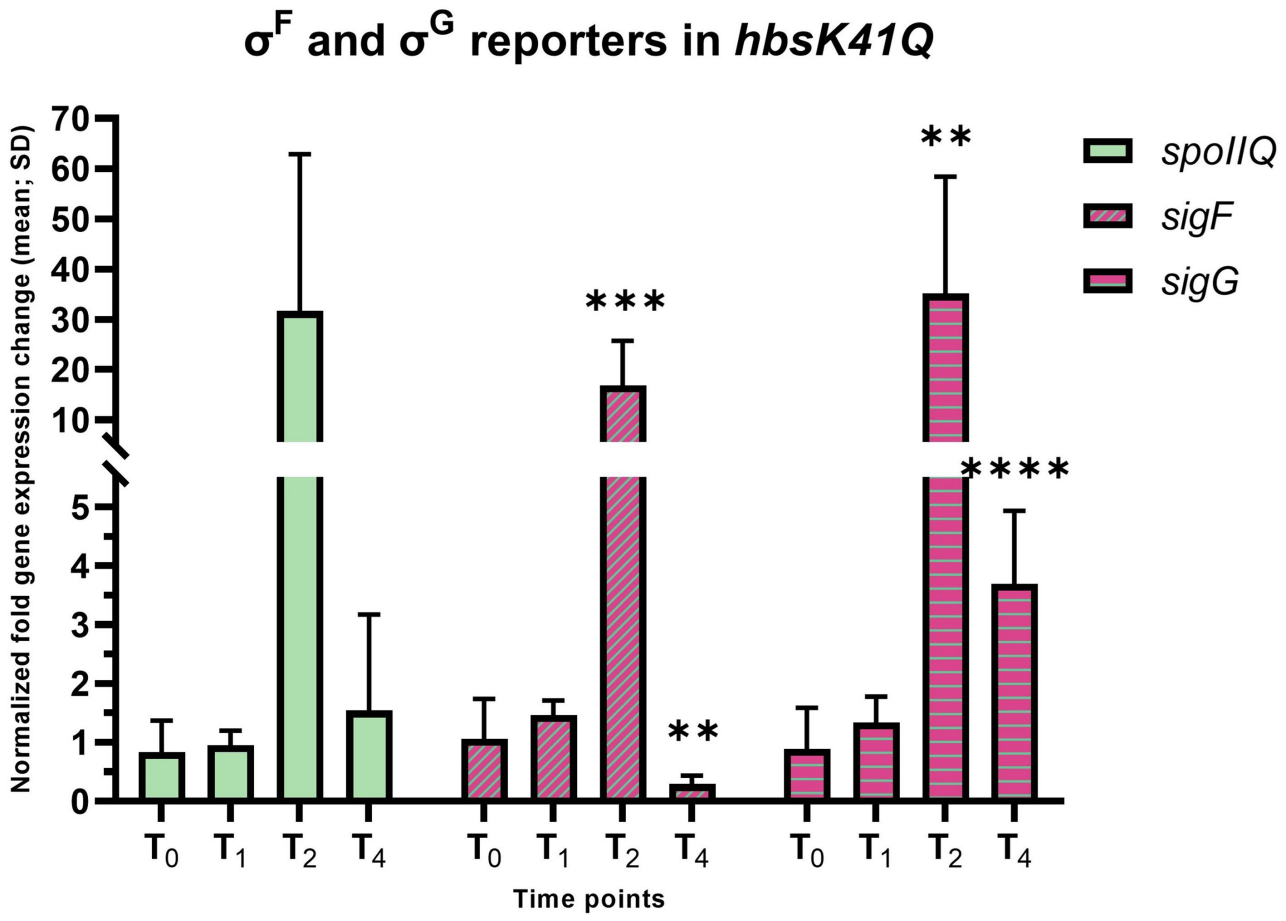
Early sporulation forespore-specific σ^F^ activity in the *hbsK41Q* strain. Wildtype and the *hbsK41Q* mutant were grown in DSM for 6 h and analyzed at time points T_0_–T_4_. Values displayed are the mean fold change values of technical duplicates from at least three biological replicates with the standard deviation (SD). Adjusted *p*-values after correction for multiple comparisons are reported, **p < 0.01, ***p < 0.001, ****p < 0.0001.

**Figure 5:**
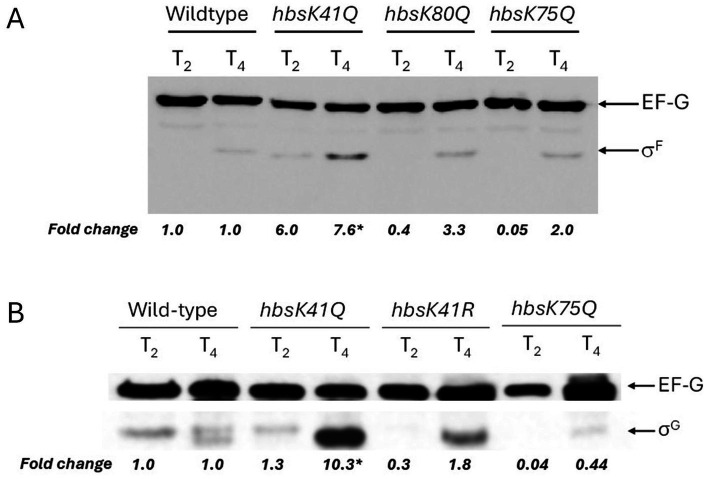
σ^F^ and σ^G^ levels in acetylation mutants. Wild-type, *hbsK41Q*, *hbsK80Q, hbsK75Q* cells were grown to T_2_ and T_4_ in sporulation media and lysates prepared as described in Materials and Methods. EF-G was included as a loading control. Non-labeled bands represent cross-reacting bands. All western blots were repeated three independent times, and a representative blot is shown. Band intensities were quantified using ImageJ, and fold changes were calculated by normalizing to EF-G and mutants compared to wildtype. **(A)** Equal amounts of protein were loaded and probed with anti-σ^F^ and anti-EF-G antibodies. **(B)** Equal amounts of protein were loaded and probed with anti-σ^G^ and anti-EF-G antibodies. The EF-G and σ^G^ blots are from the same gel with different exposure times during development. The σ^G^ signal is weaker and requires a longer exposure than EF-G, which becomes saturated. The complete, unmodified blots taken at the different exposure times are provided in Supplementary Figure S3. *p-value < 0.05.

σ^F^ and σ^E^ are compartment-specific sigma factors that are active in the forespore and mother cell, respectively. Engulfment is the morphological process following σ^E^ activation, which occurs under the transcriptional control of both σ^F^ and σ^E^. The coordinated transcription between the distinct cellular compartments leads to the completion of engulfment, generating a double-membrane organelle within the mother cell. We found that *sigF* transcription was significantly increased at T_2_ (16.83-fold, adj. *p*-value = 0.0011), and decreased at T_4_ (0.29-fold difference, adj. *p*-value = 0.0071, Figure 4). We analyzed expression levels of σ^F^ by Western blot, and found that in agreement with RT-qPCR, σ^F^ was overexpressed 6 and 7.6-fold at T_2_ and T_4_, respectively, even though transcription was reduced at T_4_ (Figure 5A). The increased protein levels, coupled with wild-type levels of the anti-sigma factor SpoIIAB (Supplementary Figure S2), implied that σ^F^ activity will also be increased, so σ^F^-dependent genes were analyzed, including *spoIIQ* and *sigG*. For *spoIIQ*, there was a 31.7-fold overexpression at T_2_, although not statistically significant, due to the presence of outliers among replicates. At T_2_ there was an overexpression of *sigG* (35.1-fold, adj. *p*-value = 0.0071) and at T_4_ there was a 3.69-fold increase (adj. *p*-value = 0.00006) in expression. In agreement, there was a 10.3-fold increase in protein at levels at T_4_ (Figure 5B). Together, these results suggest that overproduction of σ^F^ at T_2_ leads to inappropriately increased expression of the σ^F^ regulon.

**Figure 6:**
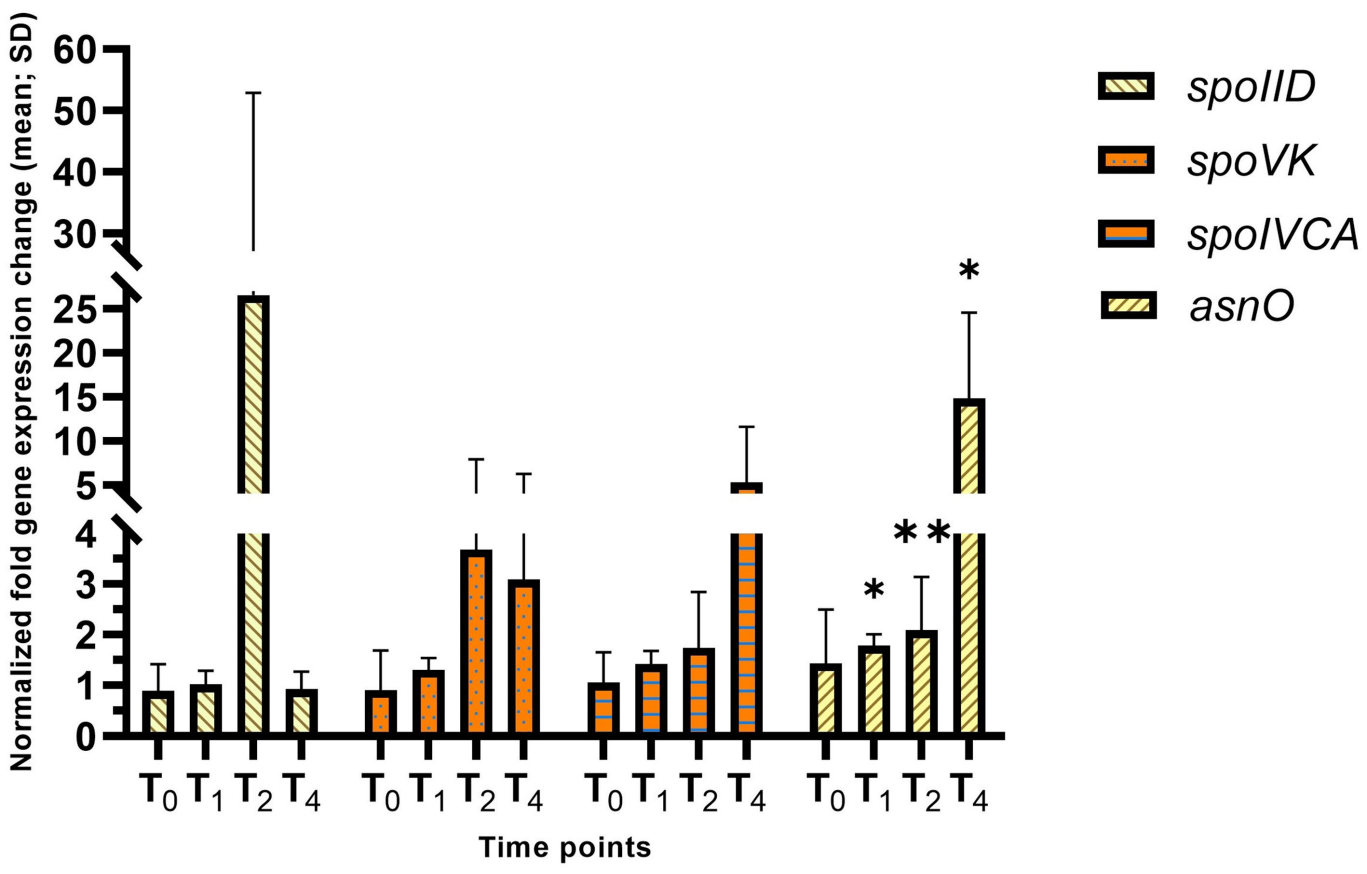
Early sporulation mother cell-specific reporters of σ^E^ activity in *hbsK41Q* strains. Wildtype and the *hbsK41Q* mutant were grown in DSM for 6 h and analyzed at time points T_0_–T_4_. Values displayed are the mean fold change values of technical duplicates from at least three biological replicates with the standard deviation (SD). Adjusted *p*-values after correction for multiple comparisons are reported, *p < 0.05, **p < 0.01.

To assay the mother-cell specific σ^E^ activity, *asnO*, *spoIID, spoIVCA,* and *spoVK* were analyzed. There was a large increase in *spoIID* levels (26.6-fold), which is only dependent upon σ^E^ for activation, suggesting increased sigma factor activity. *asnO* was significantly increased across the entire course, with a 1.79, 2.83, and 66-fold increase at T_1_, T_2_, and T_4_, respectively (adj. *p*-value = 0.031, 0.0027, and 0.0474, respectively). *spoVK* and *spoIVCA* were both overexpressed at the later time points, while not statistically significant (Figure 6). These results suggest that σ^E^ activity is also increased when K41 is acetylated.

### 3.3 Acetylation at lysine 41 increases expression of the late sporulation genes

**Figure 7:**
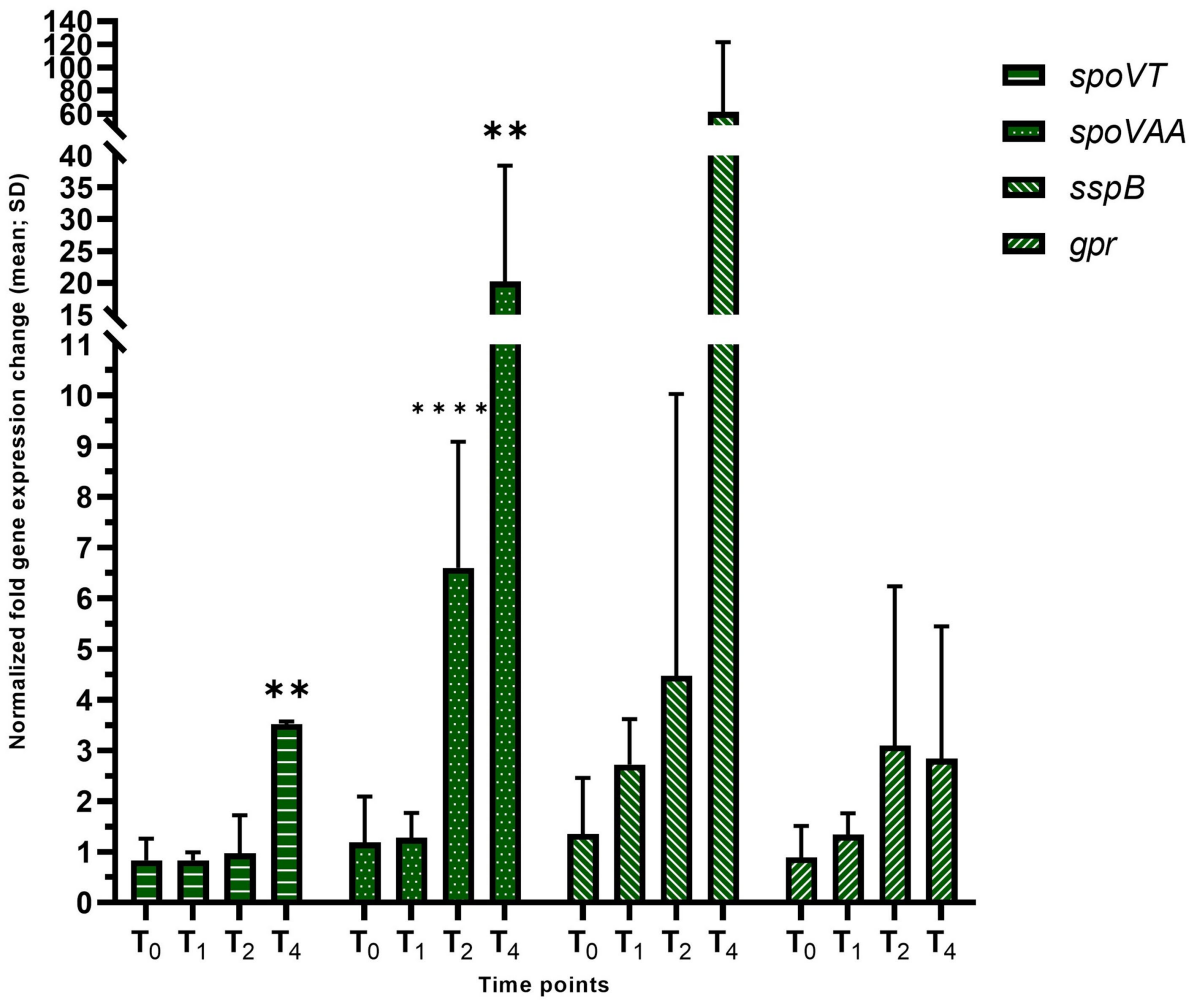
Late sporulation forespore-specific reporters of σ^G^ activity in the *hbsK41Q* mutant. Wildtype and the *hbsK41Q* mutant were grown in DSM for 6 h and analyzed at time points T_0_–T_4_. Values displayed are the mean fold change values of technical duplicates from at least three biological replicates with the standard deviation (SD). Adjusted *p*-values after correction for multiple comparisons are reported, **p < 0.01, ****p < 0.0001.

The completion of engulfment stage dramatically changes the transcriptional program within both compartments, with the activation of σ^G^ in the forespore. To assay σ^G^ activity, we examined the expression of *spoVAA*, *gpr*, *spoVT* and *sspB* (Figure 7). At the T_4_ time point *spoVT* was overexpressed 7.87-fold (adj. *p*-value = 0.002) and *sspB*, which is solely dependent on σ^G^ for expression, was overexpressed at 2.72-fold, 4.47-fold, and 61.6-fold at T_1_, T_2_, and T_4_, while not statistically significant. The presence of a large outlier in one replicate (>120-fold increase, compared to ~30-fold for all replicates) is the reason statistical significance was not reached T_4_, despite the large increase in expression. *spoVAA* was overexpressed at T_2_ (6.59-fold, adj. *p*-value = 0.000009) and T_4_ (26.8-fold, adj. *p*-value = 0.0071), whereas *gpr* did not exhibit large differences in expression.

**Figure 8:**
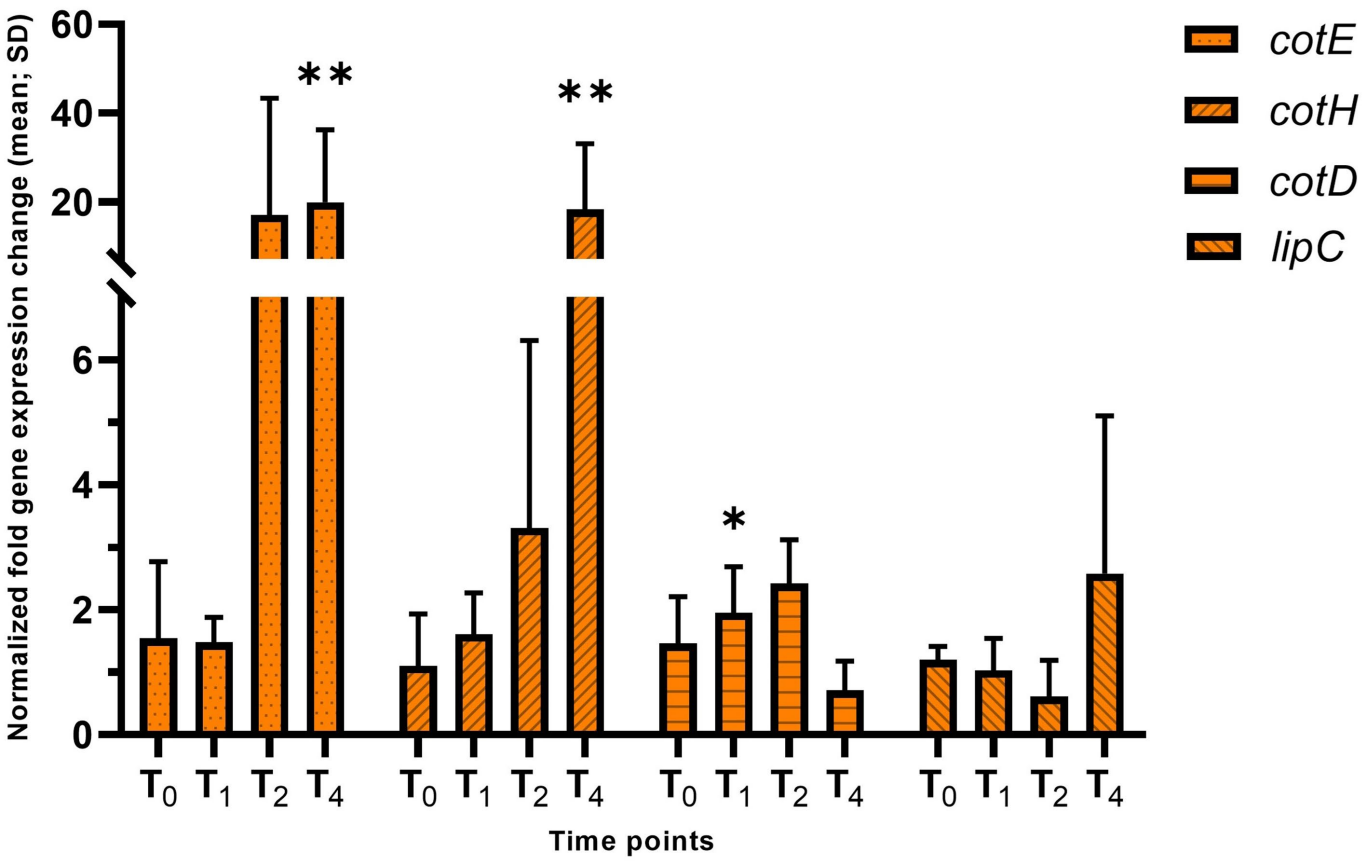
Late sporulation mother cell-specific reporters of σ^K^ activity in *hbsK41Q*. Wildtype and the *hbsK41Q* mutant were grown in DSM for 6 h and analyzed at time points T_0_–T_4_. Values displayed are the mean fold change values of technical duplicates from at least three biological replicates with the standard deviation (SD). Adjusted *p*-values after correction for multiple comparisons are reported, *p < 0.05, **p < 0.01.

**Figure 9:**
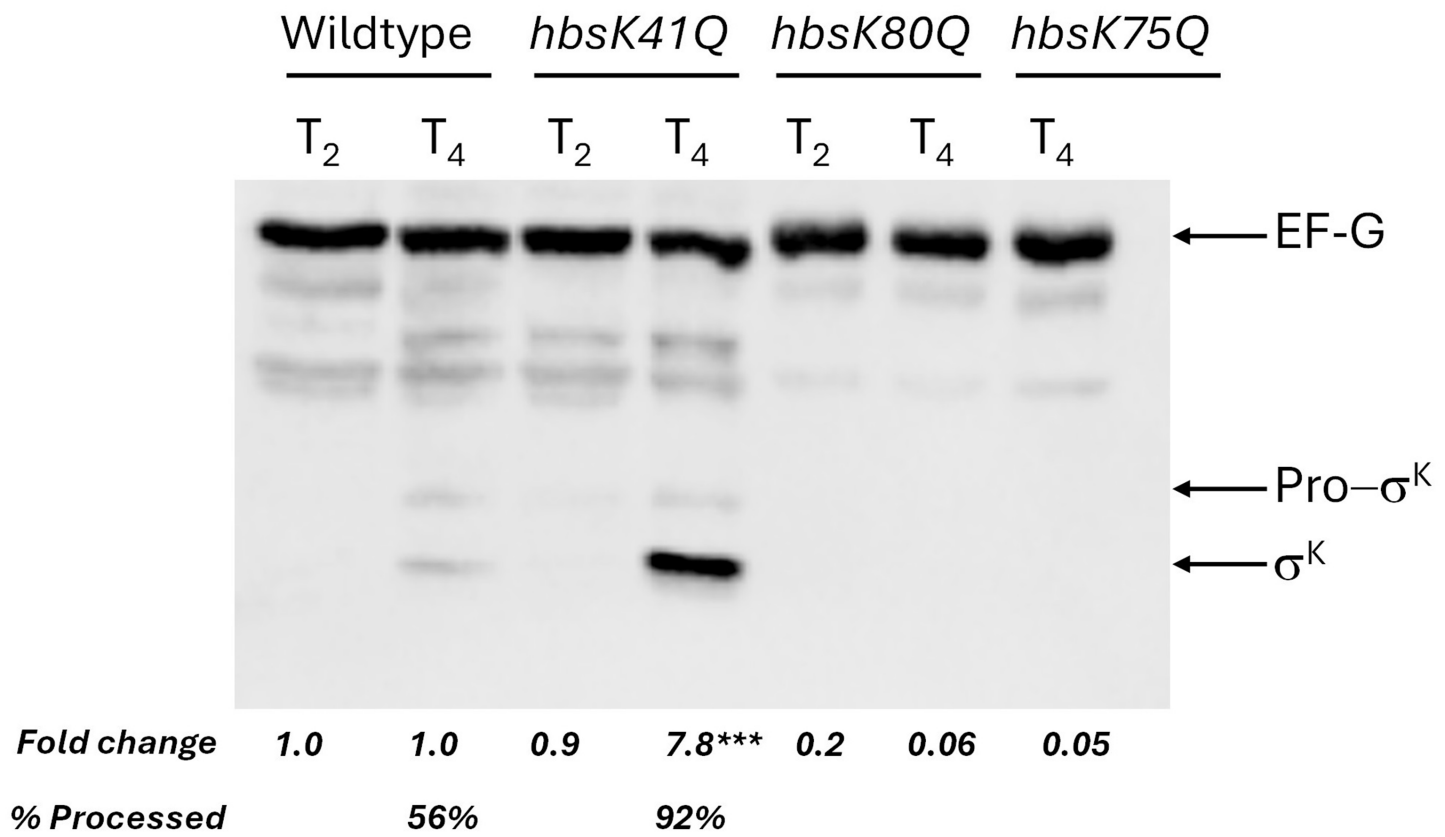
σ^K^ levels in acetylation mutants. Wild-type, *hbsK41Q*, *hbsK80Q, hbsK75Q* cells were grown to T_2_ and T_4_ in sporulation media and lysates prepared as described in Materials and Methods. Equal amounts of protein were loaded and were probed with anti-σ^K^ and anti-EFG antibodies. EFG was included as a loading control. Non-labeled bands represent cross-reacting bands. σ^K^ is detected in two forms: inactive precursor pro-σ^K^ (top band) and activated σ^K^ (bottom band). All western blots were repeated three independent times, and a representative blot is shown. Band intensities were quantified using ImageJ, and fold changes were calculated by normalizing to EF-G and mutants compared to wildtype. The values for full length σ^K^ are shown. ***p-value < 0.001.

To analyze the late stages of sporulation, we examined the expression of the coat proteins. The expression of *cotE* and *cotH* is under the control of σ^E^ and σ^K^, and *cotD* is under the control of only σ^K^. We observed a significant increase in the expression level of *cotE* (19.9-fold, adj. *p*-value = 0.0003) and *cotH* (18.3-fold, adj. *p*-value = 0.0002) at T_4_ (Figure 8). *cotD* and *lipC* expression were not different from wildtype, suggesting that σ^K^ activity was not altered. The large increase in *cotE* and *cotH* expression likely resulted from the increase in σ^E^ activity. As we observed a gradual increase in the expression of *spoIVCA* in the *hbsK41Q* mutant, which encodes a protein essential for posttranslational processing of pre-σ^K^, we examined σ^K^ levels by western blot (Figure 9). σ^K^ was overproduced at T_4_ compared to wildtype. σ^K^ is produced as a longer, inactive form that must be cleaved N-terminally for activation and both bands were detected. The higher band is the inactive protein before cleavage, and the smaller band represents the active protein. σ^K^ is overproduced at T_4_ compared to wildtype, with significantly more processing (92% compared to 56%).

### 3.4 Deacetylation of lysine 41 does not induce gene expression

Since we observed many changes associated with the *hbsK41Q* mutants, we analyzed the opposite, deacetylated form with the *hbsK41R* mutant strain. At T_2_, for most genes analyzed, similar levels to wildtype were observed (Table 2). *asnO, spo0F, spoVK* and *spoVG* were increased 2- to 6-fold in the *hbsK41Q* strain but displayed reduced expression in the *hbsK41R* strain. This suggests that many of these genes may be regulated by HBsu and acetylation at K41 is required for gene expression at T_2_. At T_4_, roughly one third of the transcripts that were significantly increased in the *hbsK41Q* strain were altered to the same extent in the *hbsK41R* strain. For example, *cotE* and *cotH* were both highly overexpressed in the *hbsK41Q* mutant (19.9-fold and 18.3-fold, respectively) and were overexpressed to a similar extent in the *hbsK41R* mutant (27.4-fold, adj. *p*-value = 0.0003 and 26.8-fold, adj. *p*-value = 0.0002, respectively). This might be due to the influence of other transcription factors and suggests that HBsu K41 acetylation is not the primary regulatory determinant for expression of these genes at T_4_.

**Table 2:** Gene expression fold change between *hbsK41Q* and *hbsK41R* mutants at T_2_ and T_4_ compared to the wildtype.

Gene	T_2_		Gene	T_4_	
	*hbsK41Q*	*hbsK41R*		*hbsK41Q*	*hbsK41R*
*spo0A*	2.62**	1.0	*spo0A*	0.51	1.0
*spo0F*	1.0	0.31	*spo0F*	0.18	0.32
*spoVG*	2.19	0.53	*kinA*	0.23	0.27
*spoIIAB*	18.4**	1.0	*sigF*	0.29**	0.56
*spoIIE*	34.1**	1.0	*spoIIAB*	0.45	1.0
*spoIIGA*	41.8**	1.0	*spoIIE*	0.45	1.0
*sigF*	16.8**	1.0	*rsfA*	4.52**	1.0
*rsfA*	0.52**	0.52**	*cotE*	19.9**	27.4**
*spoIIQ*	31.7	1.0	*cotH*	18.3**	26.8**
*spoIID*	26.6	1.52	*asnO*	72.6*	55.3
*asnO*	2.83**	0.5	*sigG*	3.69****	5.25***
*spoVK*	5.59	0.23	*spoVT*	7.87**	9.3**
*sigG*	35.1****	1.0	*spoVAA*	26.8**	1.0
*spoVAA*	6.59**	1.0	*spoVG*	1.31	0.26
*cotD*	2.42*	1.0			

Values represent average fold changes of technical duplicates from at least three biological replicates. Red font indicates cases where genes in *hbsK41R* were decreased compared to wildtype but increased in *hbsK41Q*. Green font indicates genes that were upregulated in both *hbsK41Q* and hbsK41R backgrounds. Adj. *p*-values were calculated as described in Materials and Methods. *adj. *p*-value < 0.5, **adj. *p*-value < 0.01, *** adj. *p*-value < 0.001. **** adj. *p*-value < 0.0001.

### 3.5 The acetylation state of HBsu at lysine 41 increases sporulation frequency at inappropriate times

Since most important sporulation genes were overexpressed and sigma factor activity was increased, we hypothesized that *hbsK41Q* strains sporulate earlier than the wildtype. To test this idea, we examined sporulation frequencies at T_0_ (Supplementary Figure S4), a time when the decision to enter sporulation usually occurs, and few or no intact spores should be observed. At T_0_, the wild-type and *hbsK41R* strains sporulated at a very low frequency of 0.04 and 0.07%, respectively, as expected. However, there was a 12.5-fold increase in sporulation frequency of *hbsK41Q* strains (0.55%), which agrees with the idea that increased gene expression leads to earlier sporulation. Given that *hbsK41Q* is more susceptible to heat than the wildtype, and the sporulation frequency assay utilizes a 30-min heat exposure to eliminate vegetative cells, we are likely underestimating the actual sporulation frequency. In agreement with these findings, 2-fold more *hbsK41Q* cells entered sporulation at T_0_ compared to wildtype, and by T_1_, 3-fold more cells made it to late sporulation (Supplementary Figure S1). This suggests that increased gene expression of the early sporulation genes increases the likelihood of transition into and progression through the sporulation program.

### 3.6 Acetylation of K3, K37, K75, K80, and K86 leads to a decrease in gene expression of key sporulation effectors

**Figure 10:**
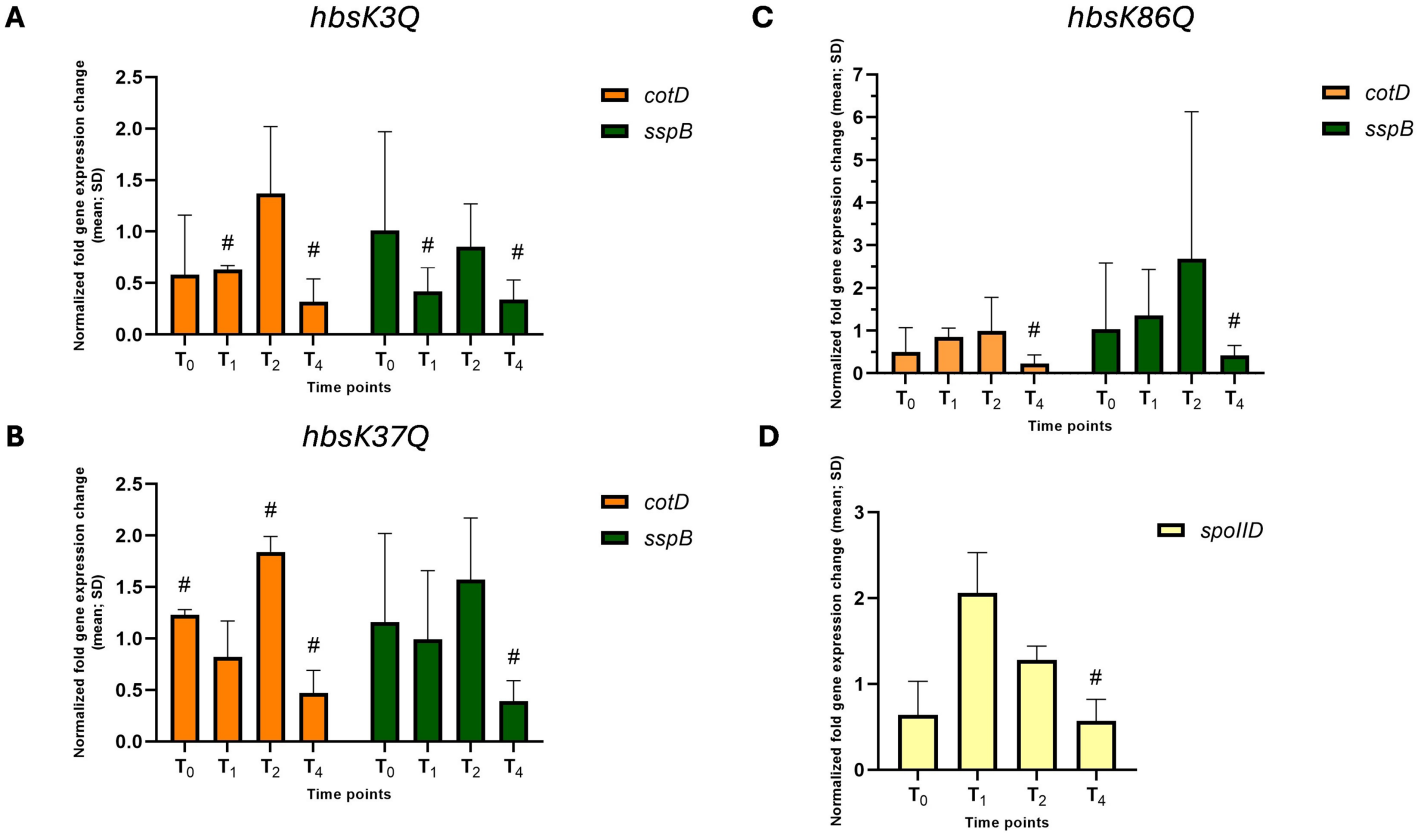
Altered gene expression in *hbsK3Q*, *hbsK37Q*, and *hbsK86Q* mutants. Wildtype and the mutants were grown in DSM for 6 h and analyzed at time points T_0_–T_4_. Values displayed are the mean fold change values of technical duplicates from at least three biological replicates with the standard deviation (SD). **(A)** *hbsK3Q* mutant, **(B)** *hbsK37Q*, **(C,D)** *hbsK86Q* mutant. ^#^Significant after univariate analysis, but not significant after correction for multiple comparisons.

Previously, we found significant reductions in sporulation frequency if the acetylation status was altered at K3, K18, K37, K75, K80, and K86 (Luu et al., 2021). For each of these sites, we observed a reduction in the expression of key genes throughout the sporulation cycle in acetylation mutants, exactly opposite to that seen in the *hbsK41Q* strain. The following results were statistically significant in univariate analyses but did not remain significant after correction for multiple testing. Nonetheless, we consider these findings biologically relevant and have chosen to report them (Supplementary Table S4). In the *hbsK3Q* strain at T_4_, the expression of two key late sporulation proteins, *cotD* and *sspB* were reduced (0.32-fold and 0.34-fold, Figure 10A and Supplementary Table S4). The *hbsK3Q* mutant has a reduced sporulation frequency, higher susceptibility to heat and formaldehyde exposure (Luu et al., 2021). Together, our data suggests when K3 is acetylated, the late sporulation genes are not turned on at the proper time, which leads to the decreased heat and chemical stress resistance. Similarly, *cotD* and *sspB* were reduced at the T_4_ in the *hbsK37Q* mutant (Figure 10B and Supplementary Table S4). The *hbsK37Q* strain was significantly more susceptible to heat exposure, with a survival rate below 10% after 30 min (Luu et al., 2021). *hbsK86Q* spores had a reduction in sporulation frequency (<50%) and were significantly more susceptible to heat exposure (Luu et al., 2021). There was also a decrease in the late sporulation genes *cotD* and *sspB* at T_4_ (Figure 10C and Supplementary Table S4). In addition, there was a decrease in *spoIID* expression at T_4_ (Figure 10D and Supplementary Table S4). This suggests that the acetylation of K3, K37, and K86 turn off gene expression of late sporulation genes. In agreement with these findings, we observed reduction in mature spores compared to wildtype at T_4_ (Supplementary Figure S1). In agreement, we observed a 2.5- to 6-fold reduction in mature spores produced at T_4_ compared to the wildtype (Supplementary Figure S1), which may indicate a defect in spore assembly and maturation.

**Figure 11:**
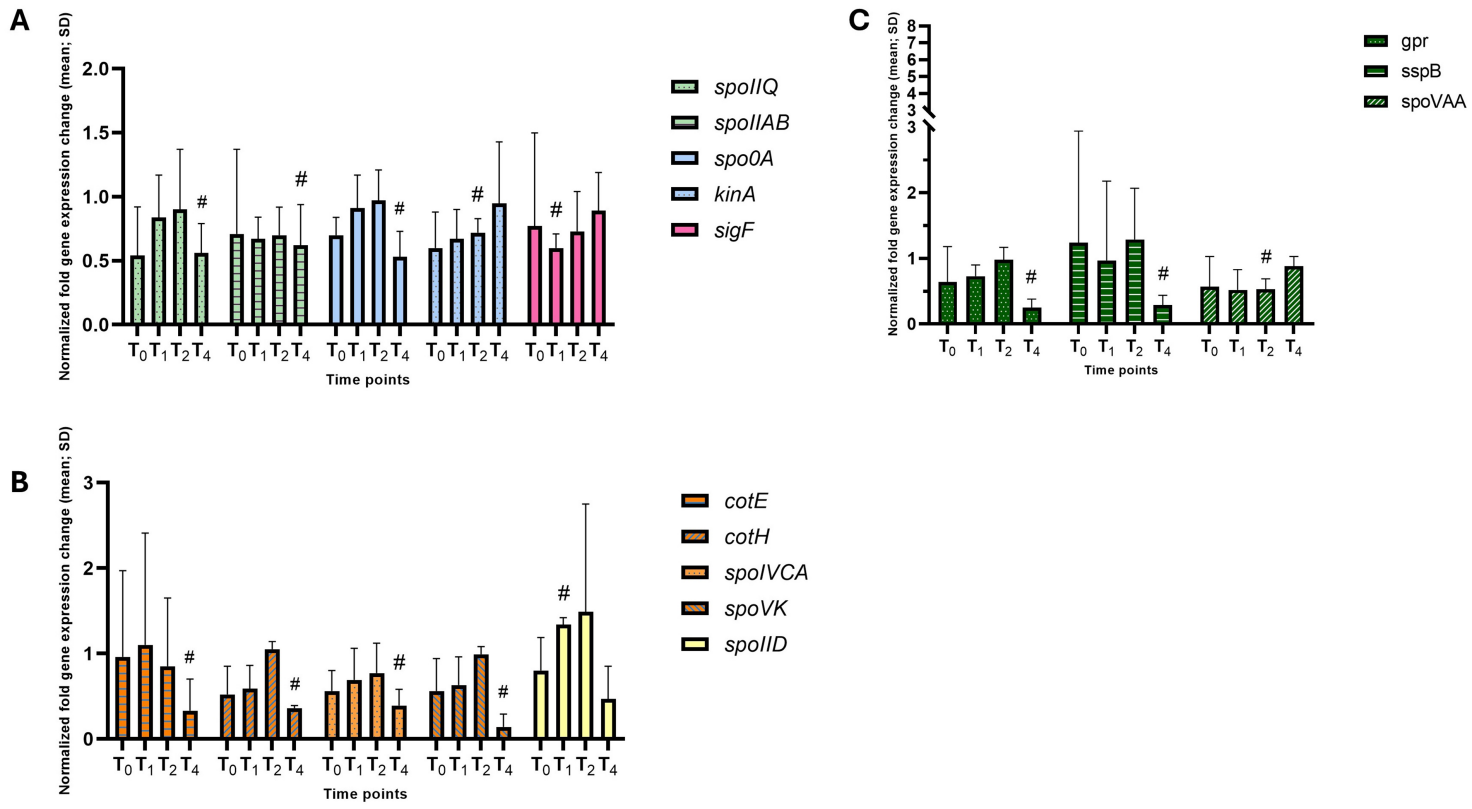
Altered gene expression in *hbsK75Q* mutants. Wildtype and the *hbsK75Q* mutant were grown in DSM for 6 h and analyzed at time points T_0_–T_4_. Values displayed are the mean fold change values of technical duplicates from at least three biological replicates with the standard deviation (SD). **(A)** Early sporulation genes dependent upon Spo0A and σ^H^, or σ^F^ for activation. **(B)** Late sporulation genes dependent upon σ^K^. **(C)** Late sporulation genes dependent upon σ^G^. ^#^Significant after univariate analysis, but not significant after correction for multiple comparisons.

The *hbsK75Q* mutants have a reduced sporulation frequency, but the resulting spores were resistant to heat and UV stress. The only noted defect was reduced survival to formaldehyde after 40 min of exposure (Luu et al., 2021). Some early sporulation genes were modestly reduced, including *kinA, sigF, spo0A, spoIIQ*, and *spoIIAB* (Figure 11A), with many of the effects observed at T_4_, when these genes would not be expected to be important. These changes likely do not explain the observed defects in sporulation frequency in this mutant. σ^F^ levels were reduced by about 50% at T_2_ compared to the wildtype (Figure 5A), while σ^G^ was reduced to 0.04 and 0.4 at T_2_ and T_4_, respectively (Figure 5B). σ^K^ levels were also reduced at T_4_, to 0.05-fold compared to wildtype (Figure 9). In addition, some late sporulation genes were reduced at T_4_, including *cotE*, *cotH*, and *sspB* (Figures 11B,C and Supplementary Table S4), which might explain reduced sporulation frequency and survival to formaldehyde, as well as a decreased production of mature spores compared to the wildtype (3% vs. 13%) at T_4_ (Supplementary Figure S1). In support of this, there were decreased mature spores produced compared to wildtype at T_4_ (Supplementary Figure S1).

**Figure 12:**
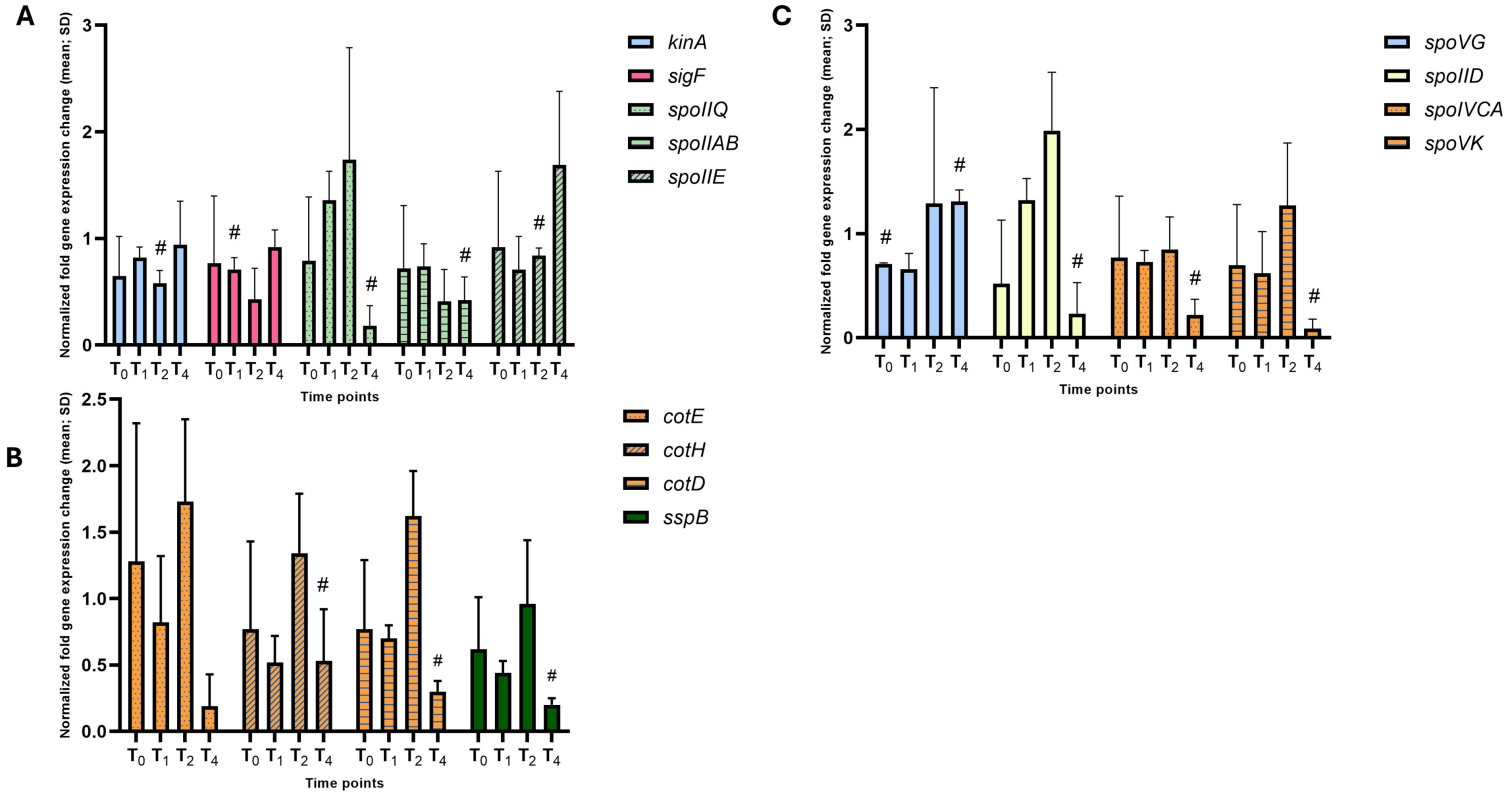
Altered gene expression in *hbsK80Q* mutants. Wildtype and the *hbsK80Q* mutant were grown in DSM for 6 h and analyzed at time points T_0_–T_4_. Values displayed are the mean fold change values of technical duplicates from at least three biological replicates with the standard deviation (SD). **(A)** Expression of early sporulation genes. Expression of σ^K^- **(B)** and σ^G^-dependent **(C)** mother-cell specific reporters at T_4_. ^#^Significant after univariate analysis, but not significant after correction for multiple comparisons.

The *hbsK80Q* mutant had the second most alterations in gene expression, with most genes, especially the mother-cell specific ones, downregulated. Previously, we found that the sporulation frequency of *hbsK80Q* strain was decreased by more than 50%, and that they were more susceptible to formaldehyde and UV radiation (Luu et al., 2021). The mature spores produced at T_4_ were reduced compared to the wildtype (2% vs. 13%), although, like the wildtype, the majority of cells proceeded to late sporulation (Supplementary Figure S1). Some forespore-specific early sporulation genes were downregulated at the T_1_ and T_2_ time points (i.e., *kinA, sigF, spoIIQ, spoIIAB, spoIIE*), which could, at least in part, explain the reduction in sporulation frequency of this mutant (Figure 12A and Supplementary Table S4). There were also reductions in σ^E^- and σ^K^-dependent genes. Most of these genes had decreased expression by more than 2-fold compared to the wildtype at the T_4_ time point (Figures 12B,C and Supplementary Table S4). Given that these late genes are involved in forming protective layers of the spores, the observed decrease in expression might explain the higher susceptibility to chemical and UV stresses. As there were multiple under-expressed genes, we next examined the levels of the sigma factors. There were no differences in σ^G^ or σ^F^ levels compared to the wildtype (Figures 5A,B). However, we were unable to detect σ^K^ (Figure 9), which might explain the reduction in the transcription of *sspB* and the *cot* genes. As acetylation of HBsu at K80 leads to decreased gene expression, this suggests that deacetylation of K80 is required for proper late gene expression.

## 4 Discussion

HBsu is a histone-like protein essential for cell viability, normal growth, and development of *B. subtilis* (Micka and Marahiel, 1992; Karaboja and Wang, 2022). Similarly to eukaryotic histones, the acetylation of HBsu decondenses chromosome by reducing its DNA-binding affinity (Klein and Marahiel, 2002; Micka and Marahiel, 1992; Carabetta et al., 2019). Previously, we found that HBsu contains seven acetylation sites (Carabetta et al., 2016). Given HBsu’s vital role in *B. subtilis* chromosome dynamics and DNA compaction, it is not surprising that HBsu was found in mature spores, at levels equal to that seen in vegetative cells (Ross and Setlow, 2000). Recently, we found that certain acetylation sites on HBsu were involved in the regulation of the process of sporulation (Luu et al., 2021). The sporulation frequency of most of the acetyl-mimicking mutants were significantly reduced, except for the *hbsK41Q* mutant (Luu et al., 2021). In addition, we found that high levels of acetylation at K75 and K80 and some intermediate levels of acetylation at K3, K41 and K86 are required for the proper protection of spores from wet heat exposure (Luu et al., 2021). We also found that acetylation at K37, K41, K75, and K80 were important for survival of chemical exposure. This work did not examine the underlying mechanism as to how HBsu acetylation influences sporulation. Based upon the known functions of HBsu, there were two likely possibilities. First, as HBsu plays a role in chromosome organization and dynamics, it might be important for axial filament formation or compaction of the chromosome into the mature spore. HBsu colocalizes with the SASPs in the mature spore and modulates SASP-mediated increases in DNA-persistence length and supercoiling (Ross and Setlow, 2000). It is possible that HBsu acetylation modulates these activities. The second possibility is that HBsu influences gene expression, by altering its DNA-binding affinity, which would change the compaction state of the chromosome and increase or decrease promoter accessibility for RNA polymerase. These possibilities are not mutually exclusive.

Here we addressed the latter possibility and examined gene expression throughout the process of sporulation to assess the impact of the acetylated forms of HBsu. Overall, all the mutants, except for *hbsK18Q*, had differences in gene expression. Acetylation at some sites changed gene expression dramatically, like *hbsK41Q* and *hbsK80Q*, although in opposite directions. These effects might have been amplified by altering the activity global regulators like the sigma factors. The most dramatic changes in the gene expression were observed in the *hbsK41Q* mutant. Many early sporulation genes (*spo0A, sigF, spoIIE, spoIIAB, spoIIQ, spoIIGA, spoIID*) and late sporulation genes (*spoVAA, sigG, cotD, spoVK, asnO*) were overexpressed at T_2_ (Figures 2–4, 6–8, respectively). Even though we examined an asynchronous population, we did not expect to find significant expression of the early sporulation genes at T_2_ because most cells have already transitioned into the sporulation program (Supplementary Figure S1). At T_4_, the late sporulation genes were highly upregulated (*cotE, cotH, sigG, spoVAA, spoVT*), possibly due to overexpression of sigma factors and transcription factors at earlier time points (Figures 7, 8). The opposite *hbsK41R* mutant did not lead to overexpression of any genes at T_2_ (Table 2), suggesting that at T_2_, acetylation at K41 activates gene expression. At T_4_, about a third of the genes were increased to a similar extent to that seen in the *hbsK41Q* strain (Table 2). It is important to note that the mutant strains represent a state with 100% acetylation or deacetylation at the specific site, whereas in nature, the process is continuous, and it is unknown if this state ever exists. Taken together, some acetylation level might be required during late sporulation at specific genes, but our data suggests that K41 should be mostly deacetylated for proper gene expression. We do not know if in wild-type cells, K41 is mostly acetylated early in sporulation to drive gene expression and becomes deacetylated at T_2_ to turn off expression. Or if under normal conditions, a low level of acetylation occurs at T_2_ to activate gene expression. In either case, it appears that acetylation at K41 serves as an “on–off” switch and may drive the transition between early and late stages of sporulation. We are currently developing a mass spectrometry-based proteomics assay to measure the *in vivo* stoichiometry of acetylation of HBsu throughout the sporulation process, which will address these possibilities. One way of controlling this transition might be through the regulation of the expression and activity of σ^F^, σ^G^, and σ^K^. We found that all three of these sigma factors were overproduced at T_4_ (Figures 5, 9), which likely disrupts the timing of the genetic program, and consequently leads to the loss of resistance properties of mature spores. In agreement, we found that *hbsK41Q* cells had increased levels of sporulation during vegetative growth, suggesting they enter the sporulation program early, even without appropriate signals (Supplementary Figure S4). The K41Q site appears to be important for stationary phase development. From our original characterization of acetylation in HBsu, we found that acetylation of K41 was the only site with significantly increased abundance in stationary phase compared to exponential phase in glucose minimal media (Carabetta et al., 2016). We also found that *hbsK41Q* mutants had larger, expanded nucleoids in response to drug challenge, which is opposite to the normal wild-type response, which is to compact the nucleoid (Carr et al., 2024). In the same study, we observed 2.26-fold increase in the formation of persisters in the *hbsK41Q* background, and a faster recovery from the persistent state, compared to the wild-type (Carr et al., 2024). We proposed that the acetylation of K41 decondenses the chromosome to allow for cells to “escape” from the persistent state and resume growth. During sporulation, the acetylation of K41 likely triggers a transition between early and late sporulation, driven by altered gene expression. Given these findings, acetylation of K41 might also regulate gene expression to allow cells to escape from a drug-induced persistent state. This possibility has yet to be determined.

For all the other mutants, except for *hbsK18Q*, there was a downregulation of sporulation-specific genes. Using a univariate statistical analysis identified significant changes in these strains, although the subsequent correction for multiple testing eliminated significance. Taking into account findings from our previous studies, we consider these findings biologically important and hypothesis generating. Acetylation of *hbsK18Q* likely does not influence gene expression, which cannot explain the observed reduction in sporulation frequency previously observed (Luu et al., 2021). It is still possible that acetylation at K18 influences chromosome dynamics or compaction of DNA into the mature spore, which will be addressed as part of a future study. The most changes were found in *hbsK80Q* and *hbsK75Q* (Figures 11, 12). The effects were largest at the later time points, especially T_4_. In the *hbsK80Q* strain, many early and late sporulation-specific genes were downregulated more than 2-fold compared to the wildtype (*cotE, spoIIQ, spoIIAB, spoIVCA, spoVK, cotD, spoIID, sspB*). A similar picture was observed in *hbsK75Q* with downregulation of *cotE, cotH, spoIIQ, spoIIAB, spoIVCA* at T_4_. This could be due to the proximity of K75 and K80 residues within the structure of the HBsu molecule. Acetylation of either site may turn off the expression of the same genes. In support of this idea, both mutants were resistant to heat but sensitive to formaldehyde exposure (Luu et al., 2021). It is possible that both sites being acetylated completely turns off gene expression, while only one leads to intermediate levels of expression, which will be further explored. There were only a few changes for *hbsK3Q, hbsK37Q,* and *hbsK86Q* strains and down regulation of *cotD and sspB* at T_4_ were common among them. These mutants were also more susceptible to heat exposure than wildtype after 20 and 30 min. Our data suggests that *sspB* and, possibly, *cotD* may contribute to heat resistance. This correlates with previously published findings on reduced resistance of *α*^−^ß^−^ SASP mutant spores to dry heat, compared to the wildtype (Setlow and Setlow, 1995). CotD assembles to the inner spore coat and spores that lacked an inner coat were extremely sensitive to lysozyme (Riesenman and Nicholson, 2000). However, the role of CotD in spore heat resistance is unknown. Downregulation of the genes encoding coat proteins in *hbsK3Q, hbsK37Q, hbsK75Q, hbsK80Q,* and *hbsK86Q* at T_4_ may partially contribute to the observed reduction of spore count in these strains (Supplementary Figure S1) and decrease in sporulation frequency. This suggests that these strains may have a spore maturation defect, which will be further explored. A complete analysis of the expression of all late structural proteins by RT-qPCR and analysis of the structure of the interior and exterior spore coat layers using transmission electron microscopy will be performed (Driks and Eichenberger, 2016).

There are two known deacetylases (KDACs) in *B. subtilis,* and we previously identified five novel HBsu acetyltransferases (KATs) (Carabetta et al., 2019; Gardner and Escalante-Semerena, 2009). In our previous study, we observed a significant decrease in sporulation frequency of the deletion mutant strains ∆*yfmK*, ∆*ydgE* and a double-mutant ∆*yfmK* ∆*ydgE*, whereas these mutations did not affect the resistance properties of the spores (Luu et al., 2021). These KATs are likely not solely responsible for acetylation at K41, as if there were true, we would expect to see no decrease in sporulation frequency, like observed in the *hbsK41R* strain (Luu et al., 2021). However, it seems like a sporulation-specific KAT exists, that is activated during early sporulation and advances the cells through the sporulation process by specifically acetylating K41, and possibly other sites. The KATs might have different target sites of HBsu, which was suggested by our previous work (Carabetta et al., 2019), but needs to be confirmed. If there were more than one KAT, they could be expressed at different times, which could help the sporulation process continue. It is also possible that they may have additional targets. If there are sporulation-induced KATs, then as the sporulation process advances, a deacetylase would have to be activated to help transition the cells between early and late stages of sporulation. These possibilities are being actively explored in the lab. Together, this data strongly supports the existence of a histone-like code in bacteria and further studies to decipher how bacteria set, erase, and read this code are warranted.

Currently, there are declining options for antibacterial treatment, and these drugs are ineffective against spores, as they are dormant and highly resilient (Butler et al., 2023; Louie et al., 2012; Romero-Rodriguez et al., 2023). Therefore, continuous research to find new targets and classes of antibiotics with mechanisms of action that could prevent sporulation or promote spore elimination is of interest (Andryukov et al., 2021). Disruption of the sporulation-specific gene expression program likely leads to asynchronicity in the intercompartment signaling, which disorganizes the transcriptional program and leads to deficiencies in the final product. We showed that the acetylation state of HBsu is essential for proper gene expression and subsequent resistance characteristics of spores. As a future direction, synthesis of the new small molecule inhibitors that would specifically target HBsu acetylation, either a specific KAT, KDAC, or HBsu itself, could become the foundation for a new class of antibacterial agents that impair sporulation frequency and spore resistance properties by altering the acetylation state of the histone-like proteins.

## Data Availability

The original contributions presented in the study are included in the article/Supplementary material, further inquiries can be directed to the corresponding author.
