# Supplementary material for: Acetylation of the histone-like protein HBsu at specific sites alters gene expression during sporulation in Bacillus subtilis
Source: Front Microbiol. 2025 Oct 23;16:1629989. doi: 10.3389/fmicb.2025.1629989 (PMC12589092; doi:10.3389/fmicb.2025.1629989)
Supplement: Supplementary file 1 [file Data_Sheet_1.docx]

**The acetylation of the histone-like protein HBsu at specific sites alters gene expression during sporulation in *Bacillus subtilis***

Liya Popova, Hritisha Pandey, Olivia R. Schreiber, Charalampos Papachristou, Valerie J. Carabetta

**Table of Contents**

[**Figure S1.** Staging of wild-type and acetylation mutant cells in DSM. 2](#_Toc210379970)

[**Figure S2.** SpoIIAB levels in wild-type and *hbsK41Q* strains. 3](#_Toc210379971)

[**Figure S3.** Unmodified versions of Western blot in figure 5 at different exposures. 4](#_Toc210379972)

[**Figure S4.** Sporulation frequences at T_0_ under sporulation conditions. 5](#_Toc210379973)

[**Table S1.** Primers used in this study 6](#_Toc210379974)

[**Table S2.** Primer efficiency for selected housekeeping genes. 8](#_Toc210379975)

[**Table S3.** List of selected genes 9](#_Toc210379976)

[**Table S4.** P-values before and after adjustment for multiple comparisons 10](#_Toc210379977)

# **Figure S1.** Staging of wild-type and acetylation mutant cells in DSM.

Wild-type and mutant cells were grown in DSM, with time points taken from T_0_-T_4_. Cells were collected, fixed, and stained with wheat germ agglutinin conjugated with Alexa Flour 488 and Sytox. Cells were seeded on a 1% agarose pad, and imaged by microscopy, as described in Materials and Methods. The staging was determined using biological replicates, analyzing at least 200 cells in each sample. Early refers to sporulation stages I-III, late refers to stages IV-VI, and spores refers to the presence of phase-bright, mature spores. Note that for *hbsK41R* strains, there was a 2-fold increase in mature spores identified at T_0_ compared to wildtype. While we do not know the exact reason for this observation, it is possible that these spores exhibit a germination defect and remain following dilution into fresh DSM. At early timepoints, *hbsK41Q* cells (red, bold) may be more likely to enter the sporulation program, due to increased gene expression.

# **Figure S2.** SpoIIAB levels in wild-type and *hbsK41Q* strains.


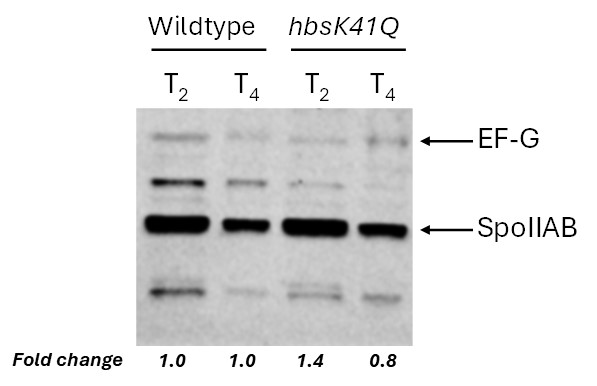


Wild-type and *hbsK41Q* cells were grown to T_2_ and T_4_ in sporulation media and lysates prepared as described in Materials and Methods. Equal amounts of protein were loaded and were probed with anti-SpoIIAB and anti-EF-G antibodies. EF-G was included as a loading control. Non-labeled bands represent cross-reacting bands. All western blots were repeated three independent times, and a representative blot is shown. No significant differences were found.

# **Figure S3.** Unmodified versions of Western blot in figure 5 at different exposures.


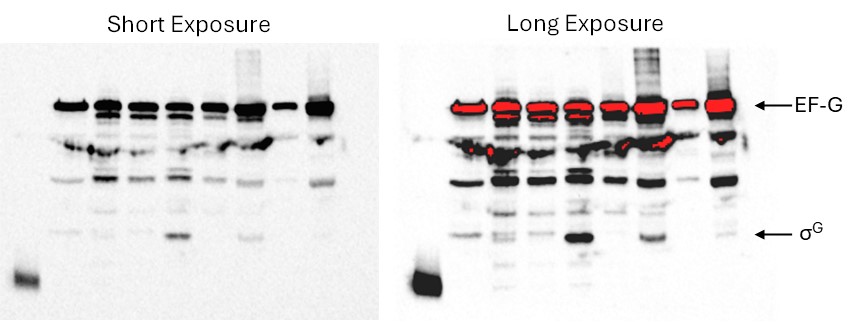


Full, uncropped versions of the western blot in Fig. 5. The short exposure was for 15 sec, which was necessary to visualize the EF-G signal. The longer exposure was for 60 sec to visualize the σ^G^ band. The instrument false-colored bands red that were saturated and outside of the linear range of detection. The EF-G band is saturated at this exposure, necessitating analysis at different exposure times for each signal. Unlabeled bands represent non-specific cross-reacting bands.

# **Figure S4.** Sporulation frequences at T_0_ under sporulation conditions.

Wild-type, *hbsK41Q,* and *hbsK41R* cells were grown for 2 hours in DSM media to T_0_. The displayed values are the mean of three biological replicates, with standard deviation shown. For the wild-type and *hbsK41R* strains, there was little sporulation, but detectable levels of sporulation in the *hbsK41Q* strain. None of the differences were statistically significant.

# **Table S1.** Primers used in this study

| **Primer name** | **Sequence (5’ 🡪 3’)** |
| --- | --- |
| 5-cotD-RT | TGGCGCCAATTGTCCATCCT |
| 3-cotD-RT | ACGTGCTGAAAATGCTGGTGG |
| 5-spoIID-RT | CAGTCAGCACGAAACCAGCA |
| 3-spoIID-RT | AGGCGACGACTCCAATCACA |
| 5-sspB-RT | ACCTTGGAGCGGACACAACT |
| 3-sspB-RT | TGAACTCTGCCGCCCATTTG |
| 5-spoVG-RT | CGATTGCATCCATCACGCTG |
| 3-spoVG-RT | ATCAGGGGTGCGTTTACTCG |
| 5-spo0F-RT | TGGACATGAAAATTCCCGGCA |
| 3-spo0F-RT | AGAGCGCCCAATTCCTTCGA |
| 5-spo0A-RT | TCCGCCATGCAATTGAAGTGG |
| 3-spo0A-RT | ACCTCAGCTTATCCGCAACCA |
| 5-kinB-RT | ACCTGCGATTCTTGTGCCAA |
| 3-kinB-RT | AACGGGAATCATCTGAAGGCC |
| 5-spoIIGA-RT | GGAACAGCCGAAACGATGATCA |
| 3-spoIIGA-RT | TTGCTGACCAACTCCCCTGT |
| 5-spo0B-RT | TCATCTGCTTGGCCATTCCC |
| 3-spo0B-RT | GCTTTGATTCGTGCTTTGCGT |
| 5-kinA-RT | TGAGCGGACAGAACGGGAAA |
| 3-kinA-RT | CGGGCTTGCAGGTTCGATTT |
| 5-spoIIE-RT | TTCCGGGAACTGTCGAGCAT |
| 3-spoIIE-RT | GCTTTCAGACAGCGCGTGAA |
| 5-spoIIAB-RT | AGCTGGACCCGACAATGGAT |
| 3-spoIIAB-RT | ACGACATGATCTTCCAGCGTCA |
| 5-sigF-RT | GCGTCTTGTTTGGTCTGTCGT |
| 3-sigF-RT | TCGGCACTGCATACGTTGAA |
| 5-rsfA-RT | TCCGTGCTTGAGAGTGATGC |
| 3-rsfA-RT | GGATCACCGTTCGCCACAAT |
| 5-sigG-RT | AGAGGGCATGAGAAGGCTGA |
| 3-sigG-RT | TGGACACCTGCGCTTGAGAA |
| 5-gpr-RT | TGACACCTGATGCGCTTGGA |
| 3-gpr-RT | TGCAAAAGCACTGACAGGCC |
| 5-spoIIQ-RT | TAGTCAGTGCGGCCGTCATT |
| 3-spoIIQ-RT | ACTGCATCGTCGTTGTTGTCA |
| 5-spoVK-RT | ACACCGCCCAAAAGACGAGA |
| 3-spoVK-RT | AAGTCCTTTTCGCCGCCTCT |
| 5-asnO-RT | TGTTGTTTGCCGCGAGAGAC |
| 3-asnO-RT | TATCAGGGTGCGCGAGGATT |
| 5-spoIVCA-RT | TCGACCGAGGAACAAGCGAT |
| 3-spoIVCA-RT | GCGATTCAAAGCCGGACGTT |
| 5-divIC-RT | CCAGGGAACGAACGATAACTGA |
| 3-divIC-RT | ACTAGGGCGCCGAATACAGT |
| 5-spoVAA-RT | TGCCGCTTTATCAGGTGAGC |
| 3-spoVAA-RT | ACAATGGTTTCTGCTCCGCC |
| 5-spoVT-RT | TCCGTGCTTGAGAGTGATGC |
| 3-spoVT-RT | GGATCACCGTTCGCCACAAT |
| 5-cotE-RT | AACCGAGCAGCATTTTGGGT |
| 3-cotE-RT | TGTCTTTGTGTTGTCCGCGT |
| 5-cotH-RT | TTTCTCGGAGCTAGGGACACTG |
| 3-cotH-RT | GCCAGCTTCCTTTTCGCCAA |
| 5-lipC-RT | AAATCCTTCCCCGTACGCCA |
| 3-lipC-RT | CCGCGATTTTGACATGGGGT |
| 5-rpoD-RT | AAAACGGTATGTCGGACGCG |
| 3-rpoD-RT | ATCGCCTGTCTGATCCACCA |
| 5-rpoA-RT | TGCTCAAAGAGGACGTGGGT |
| 3-rpoA-RT | TGCAACTTGGCCTACACGAG |
| 5-rrnA-16S-RT | TGGTTGTCGTCAGCTCGTGT |
| 3-rrnA-16S-RT | TTGTCACCGGCAGTCACCTT |
| 5-rrnA-5S-RT | TTTGGTGGCGATAGCGAAG |
| 3-rrnA-5S-RT | TCCTACTCTCACAGGGGGAAA |

# **Table S2.** Primer efficiency for selected housekeeping genes.

| Gene | Efficiency | Curve | R^2^ |
| --- | --- | --- | --- |
| *rpoA* | **79.57%** | -3.94x + 19.8 | 0.9991 |
| *rpoD* | **96.58%** | -3.53x + 23.7 | 0.9962 |
| *rrnA-16S* | **81.68%** | -3.85x + 12.3 | 0.9898 |
| *rrnA-5S* | **54.01%** | -5.33x + 19.5 | 0.9874 |

# **Table S3.** List of selected genes

| Gene | Description* | Function* |
| --- | --- | --- |
| spo0A | Sporulation transcription factor Spo0A, a phosphorelay regulator, phosphorylated in response to complex YlbF/YmcA/YaaT. | Initiation of sporulation |
| spo0B | Sporulation initiation phosphotransferase Spo0B | Initiation of sporulation |
| spo0F | Phosphotransferase, initiation of sporulation phosphorelay | Initiation of sporulation |
| kinA | Sporulation-specific, ATP-dependent protein histidine kinase, phosphorylates Spo0F | Initiation of sporulation |
| kinB | Sporulation sensor histidine kinase KinB, phosphorylates Spo0F | Initiation of sporulation |
| spoIIQ | Forespore protein, part of the transmembrane channel linking the mother cell and the forespore, stage II sporulation protein SpoIIQ | Forespore encasement by the spore coat |
| spoIIE | SpoIIAA-phosphate serine phosphatase, stage II sporulation protein E | Control of s^F^ activity, required for formation of the asymmetric septum |
| sigF | RNA polymerase sporulation-specific sigma factor (sigma-F) | transcription of sporulation genes (early forespore) |
| spoIIAB | Anti-sigma factor (antagonist of s^F^) and serine kinase | control of s^F^ activity; phosphorylation and inactivation of SpoIIAA |
| rsfA | Prespore-specific transcription regulator RsfA of s^F^ activity | Control of expression of s^F^-dependent genes |
| sigG | RNA polymerase sporulation-specific sigma factor (sigma-G) | Transcription of sporulation genes (late forespore) |
| spoIIGA | Protease processing pro- s^E^ | Maturation of s^E^ |
| spoIID | Lytic transglycosylase | Dissolution of the septal cell wall |
| divIC | Cell-division initiation protein | Septum formation |
| spoIVCA | Site-specific DNA recombinase required for creating the *sigK* gene | Excision of the Skin element, creation of the *sigK* gene |
| asnO | Asparagine synthetase (sporulation related) | Biosynthesis of asparagine |
| cotE | Morphogenic spore protein, outer spore coat protein CotE | Assembly of the outer spore coat |
| cotH | Spore coat protein kinase | Protection of CotU and CotC in the mother cell |
| cotD | Spore coat protein (inner) | Resistance of the spore |
| spoVAA | Stage V sporulation protein AA | Spore maturation |
| spoVT | Transcriptional regulator of sporulation / germination, stage V sporulation protein T | regulation of forespore gene expression |
| spoVK | Mother cell sporulation ATPase, stage V sporulation protein K | Spore maturation |
| spoVG | RNA-binding regulatory protein, affects asymmetric septation and cortex formation | Cell division, control of sporulation initiation, cortex formation |
| sspB | Small acid-soluble spore protein (major beta-type SASP) | Protection of spore DNA |
| lipC | Spore coat phospholipase B | Spore germination |
| gpr | Spore germination protease, GPR endopeptidase | Degradation of SASPs |

*Descriptions and functions are from *Subtiwiki* (Elfmann C, Dumann V, van den Berg T, Stülke J. A new framework for SubtiWiki, the database for the model organism *Bacillus subtilis*. Nucleic Acids Res. 2025 Jan 6;53(D1):D864-D870. doi: 10.1093/nar/gkae957).

# **Table S4.** P-values before and after adjustment for multiple comparisons

| Strain | *gene* | *timepoint* | *p-value nonadjusted* | *p-value adjusted* |
| --- | --- | --- | --- | --- |
| hbsK41Q | *spo0A* | T2 | 0.0000005 | 0.0011 |
|  | *spoIIAB* | T2 | 0.0173 | 0.0074 |
|  | *spoIIGA* | T1 | 0.0398 | 0.0071 |
|  | *spoIIE* | T2 | 0.0094 | 0.0018 |
|  | *sigF* | T2 | 0.0073 | 0.0011 |
|  | *sigF* | T4 | 0.0001 | 0.0071 |
|  | *sigG* | T2 | 0.0157 | 0.00006 |
|  | *sigG* | T4 | 0.0029 | 0.00006 |
|  | *asnO* | T1 | 0.0218 | 0.0312 |
|  | *asnO* | T2 | 0.0166 | 0.0027 |
|  | *asnO* | T4 | 0.0432 | 0.0475 |
|  | *rsfA* | T2 | 0.0033 | 0.0036 |
|  | *rsfA* | T4 | 0.0075 | 0.0002 |
|  | *spoVAA* | T2 | 0.0013 | 0.000009 |
|  | *spoVAA* | T4 | 0.0126 | 0.0071 |
|  | *spoVG* | T1 | 0.0034 | 0.0001 |
|  | *spoVT* | T4 | 0.0193 | 0.0019 |
|  | *cotD* | T1 | 0.1707 | 0.0382 |
|  | *cotE* | T4 | 0.0367 | 0.0003 |
|  | *cotH* | T4 | 0.0354 | 0.0002 |
| hbsK41R | *sigG* | T4 | 0.0041 | 0.00006 |
|  | *sigF* | T4 | 0.0305 | 0.4592 |
|  | *rsfA* | T2 | 0.0032 | 0.0036 |
|  | *spoVT* | T4 | 0.0276 | 0.0019 |
|  | *spoVK* | T2 | 0.0004 | 0.9988 |
|  | *cotE* | T4 | 0.0001 | 0.0003 |
|  | *cotH* | T4 | 0.0008 | 0.0002 |
| hbsK37Q | *cotD* | T0 | 0.0021 | 0.9924 |
|  | *cotD* | T2 | 0.0040 | 0.3063 |
|  | *cotD* | T4 | 0.0173 | 0.0502 |
|  | *sspB* | T4 | 0.0289 | 1.0000 |
| hbsK80Q | *spoIIQ* | T4 | 0.0001 | 0.6165 |
|  | *kinA* | T2 | 0.0104 | 0.5928 |
|  | *spoIIAB* | T4 | 0.0008 | 0.2569 |
|  | *spoIIE* | T2 | 0.0489 | 1.0000 |
|  | *sigF* | T1 | 0.0363 | 0.3133 |
|  | *cotE* | T4 | 0.0004 | 0.9999 |
|  | *cotH* | T4 | 0.0456 | 1.0000 |
|  | *cotD* | T4 | 0.0202 | 0.0109 |
|  | *sspB* | T4 | 0.0001 | 1.0000 |
|  | *gpr* | T4 | 0.0000 | 0.732 |
|  | *spoVG* | T0 | 0.0000 | 0.9999 |
|  | *spoVG* | T4 | 0.0100 | 0.8355 |
|  | *spoIID* | T4 | 0.0202 | 0.0514 |
|  | *spoIVCA* | T4 | 0.0069 | 0.9987 |
|  | *spoVK* | T4 | 0.001 | 0.9474 |
| hbsK86Q | *spoIID* | T1 | 0.0601 | 0.0374 |
|  | *spoIID* | T4 | 0.0438 | 0.3573 |
|  | *cotD* | T4 | 0.0048 | 0.0064 |
|  | *sspB* | T4 | 0.0457 | 1.0000 |
| hbsK75Q | *spo0A* | T4 | 0.0021 | 0.9147 |
|  | *spoIIQ* | T4 | 0.0067 | 0.9554 |
|  | *spoIIAB* | T4 | 0.0347 | 0.7027 |
|  | *cotE* | T4 | 0.0069 | 0.9999 |
|  | *cotH* | T4 | 0.0020 | 1.0000 |
|  | *kinA* | T2 | 0.031 | 0.8835 |
|  | *sigF* | T1 | 0.0183 | 0.1008 |
|  | *spoIVCA* | T4 | 0.0222 | 0.9840 |
|  | *spoVK* | T4 | 0.0075 | 0.9999 |
|  | *spoIID* | T1 | 0.0088 | 0.6459 |
|  | *gpr* | T4 | 0.0023 | 0.9999 |
|  | *sspB* | T4 | 0.0073 | 1.0000 |
|  | *spoVAA* | T2 | 0.0271 | 0.9941 |
| hbsK3Q | *cotD* | T1 | 0.0002 | 0.5543 |
|  | *cotD* | T4 | 0.0086 | 0.014 |
|  | *sspB* | T1 | 0.0450 | 0.9347 |
|  | *sspB* | T4 | 0.0223 | 1.0000 |
